# A novel ferroptosis inhibitor, Thonningianin A, improves Alzheimer's disease by activating GPX4

**DOI:** 10.7150/thno.98172

**Published:** 2024-09-23

**Authors:** Yuanyuan Yong, Lu Yan, Jing Wei, Chi Feng, Lu Yu, Jianming Wu, Minsong Guo, Dongsheng Fan, Chonglin Yu, Dalian Qin, Xiaogang Zhou, Anguo Wu

**Affiliations:** 1 Sichuan Key Medical Laboratory of New Drug Discovery and Drugability Evaluation, Luzhou Key Laboratory of Activity Screening and Druggability Evaluation for Chinese Materia Medica, Key Laboratory of Medical Electrophysiology of Ministry of Education, School of Pharmacy, Southwest Medical University, Luzhou, China, 646000.; 2 Department of Cardiology, The Affiliated Hospital of Southwest Medical University, Luzhou, Sichuan, China, 646000.; 3 School of Ophthalmology, Chengdu University of Traditional Chinese Medicine, Chengdu, China, 610075.; 4 Department of Pharmacy, First Affiliated Hospital of Guizhou University of Traditional Chinese Medicine, Gui Yang, China, 550000.

**Keywords:** Ferroptosis, GPX4 activation, Alzheimer's disease, thonningianin A, AMPK/Nrf2/GPX4

## Abstract

**Background:** Ferroptosis, a recently unveiled iron-dependent form of cellular demise, has emerged as a pivotal process contributing to the pathology of Alzheimer's Disease (AD). Glutathione Peroxidase 4 (GPX4), a vital defense mechanism countering ferroptosis by nullifying lipid peroxides and maintaining cellular redox equilibrium, has garnered significant attention in AD. Thus, identifying ferroptosis inhibitors to target GPX4 activation may help mitigate neuronal damage and impede AD progression.

**Objectives:** We aimed to screen potent ferroptosis inhibitors and investigate their mechanism of action and therapeutic potential in AD, as well as lay the groundwork for future research in this promising area of study.

**Methods:** This study employed a natural compound library to screen potential ferroptosis inhibitors in RAS-selective lethal compounds 3 (RSL-3)-induced PC-12 cells. Ferroptosis was evaluated by examining the mitochondrial morphology and function, reactive oxygen species (ROS) production, and lipid peroxide levels. The ability to chelate iron and intracellular iron levels was determined by UHPLC-Q/TOF-MS/MS and PGSK staining, respectively. APP Swe/ind- or Tau P301L-overexpressing PC-12 cells, and Amyloid-β transgenic CL4176 and Tau transgenic BR5270 *Caenorhabditis elegans* were employed as cellular and animal models of AD.

**Results:** Thonningianin A (ThA) was identified as a novel ferroptosis inhibitor, as demonstrated by augmented cellular viability, mitigated mitochondrial impairment, diminished lipid peroxides, iron levels, and ROS generation. Mechanistically, ThA binds with GPX4 and enhances the AMPK/Nrf2 signaling pathway to stimulate GPX4 activation, effectively inhibiting ferroptosis. Moreover, in cellular and *Caenorhabditis elegans* AD models, ThA substantially inhibits ferroptosis by reducing ROS, lipid peroxide generation, and iron accumulation. Furthermore, ThA significantly delays paralysis, ameliorates food-sensing deficits and increases worms' antioxidative capacity.

**Conclusion:** ThA ameliorates AD by inhibiting neuronal ferroptosis mediated by GPX4 activation through its binding with GPX4 and the upregulation of the AMPK/Nrf2/GPX4 pathway.

## Introduction

Alzheimer's disease (AD), the most prevalent age-related neurodegenerative disorder, is characterized by the accumulation of Amyloid-β (Aβ) senile plaques outside neurons and neurofibrillary tangles (NFTs) of hyperphosphorylated Tau protein within neurons 1, 2. These pathological hallmarks are associated with cognitive decline and diminished self-care capabilities in affected individuals 3. Despite the availability of clinical treatments, current strategies primarily offer symptomatic relief without significantly halting the progression of AD 4, 5. Consequently, the quest for therapeutic agents against AD has become a critical focus of international scientific research.

Recent evidence has underscored a significant connection between AD pathology and neuronal ferroptosis, a form of iron-dependent programmed cell death characterized by the excessive production of lipid peroxides 6-9. Elevated iron levels in the hippocampus, cortical lobe, and basal ganglia correlate with Aβ plaque accumulation, suggesting a pivotal role of iron in AD pathology 10. Additionally, characteristics of ferroptosis, including disrupted iron metabolism, glutamate excitotoxicity, and lipid reactive oxygen species (ROS) accumulation, have been observed in humans and AD animal models 11-14. The enzyme glutathione peroxidase 4 (GPX4), crucial for neutralizing lipid peroxides and supporting cellular antioxidant defense, has attracted attention for its potential in AD treatment due to its role in regulating ferroptosis 15. Studies indicate that GPX4 deficiency exacerbates neuronal death via ferroptosis in AD while enhancing GPX4 activity offers protection by reducing lipid peroxidation and preserving neuronal integrity 16, 17. Recent reports have underscored the importance of GPX4 activation and its regulation via the Nrf2 pathway in mitigating ferroptosis and AD pathology 18, 19. Moreover, treatments targeting ferroptosis, including the iron chelator deferoxamine 20 and lipid peroxide inhibitors like ferrostatin-1 (Fer-1) and liproxstatin-1 (Lip-1), have demonstrated beneficial effects on cognitive functions and neuronal health in AD models 12, 21. These findings highlight the importance of iron-dependent ferroptosis in AD progression and suggest that chelating iron and modulating GPX4 activity offer novel therapeutic avenues.

In the search for new AD treatments, an increasing number of compounds, including natural products, present a vast source of ferroptosis inhibitors 22-24. By rigorously screening a diverse library of natural substances, we identified thonningianin A (ThA) as a potent ferroptosis inhibitor. ThA demonstrated an ability to enhance cellular viability, mitigate mitochondrial dysfunction, regulate iron homeostasis, and decrease lipid peroxide levels and ROS production. Its mechanism involves binding to GPX4 and boosting the AMP-activated protein kinase/Nuclear factor erythroid 2-related factor 2 (AMPK/Nrf2) signaling pathway, leading to GPX4 activation and effective ferroptosis suppression. Notably, ThA demonstrated considerable inhibition of ferroptosis in AD models using *Caenorhabditis elegans* (*C. elegans*), which delayed the onset of paralysis, improved deficits in sensing food and boosted antioxidative capabilities. These effects are linked to the activation of *aak-2* (the equivalent of human AMPK in *C. elegans*) and *skn-1* (comparable to mammalian Nrf2). These promising results advocate for the development of ThA as a novel AD treatment strategy focused on ferroptosis inhibition.

## Materials and Methods

### Chemicals, plasmids, and antibodies

The collection of natural compounds was obtained from Herbest Bio-Tech Co., Ltd. (Baoji, China) and Push Bio-Tech Co., Ltd. (Chengdu, China). Aβ1-42 was purchased from Chinapeptides (Shanghai, China). RSL-3 and ammonium ferric citrate (AFC) were purchased from Selleckchem (TX, USA), and erastin was procured from MedChemexpress (NJ, USA). MCC950 (MCC), nigericin, necrosulfonamide (NSA), and Z-VAD-FMK (ZVF) were purchased from (GlpBio, USA). Iron (II) sulfate heptahydrate (FeSO_4_.7H_2_O) was purchased from Sigma-Aldrich (Shanghai, China). Lip-1, Fer-1, DFO, hemin, oligomycin A (OA), compound C (CC), bafilomycin A1 (Baf), 3-methyladenine (3-MA), necrostatin-1 (Nec-1), and ML385 were obtained from TargetMol (Shanghai, China). Cell culture reagents such as Dulbecco's Modified Eagle Medium (DMEM), penicillin-streptomycin, and trypsin-EDTA were secured from Gibco (CA, USA) and Biosharp (Hefei, China). Fetal bovine serum (FBS) was sourced from Procell (Wuhan, China). The GPX4 protein was obtained from TargetMol (Shanghai, China). The biotinylatign kit was obtained from Genemore (Jiangsu, China). HEPES buffer was purchased from Solarbioz. The plasmids pRK5-EGFP-Tau P301L and pEGFP-N1-APP were kindly provided by Karen Ashe, and Zita Balklava & Thomas Wassmer, respectively. Both a scrambled shRNA (pLKO.1-EGFP-Puro; #P1717) and an shRNA targeting GPX4 (pLKO.1-U6-GPX4(human)-shRNA3-CMV-EGFP-hPGK-Puro; #P23908) were developed by Miaolingbio (Wuhan, China). For this study, antibodies were utilized as follows: From ABclonal (Wuhan, China), ferritin heavy chain (FTH) (#A19544), ferritin light chain (FTL) (#A1768), glutamate-cysteine ligase complex (GCLC) (#A1038), SLC7A11 (#A2413), and GPX4 (#A11243). Nrf2 (#M200-3) was sourced from MBL (Nagoya, Japan). Cell Signaling Technology (MA, USA) supplied Keap1 (#8047S), phosphorylated AMPK (p-AMPK) (#2535L), while Proteintech (Wuhan, China) provided AMPK (#66536-1-Ig), heme oxygenase-1 (HO-1) (#10701-1-AP), NAD(P)H quinone oxidoreductase 1 (NQO-1) (#11451-1-AP), GAPDH (#60004-1-Ig), and Lamin-B1 (#66095-1-Ig). Additionally, 4-hydroxynonenal (4-HNE) (#Ab46545) was obtained from Abcam (MA, USA), and β-actin (#K2818) from Santacruz (TX, USA). The authenticity and specificity of all antibodies have been confirmed by their respective suppliers.

### Cell culture

PC-12 and SH-SY5Y cell lines, obtained from the American Type Culture Collection (ATCC, USA), were grown in DMEM enriched with 10% FBS, 50 U/mL of penicillin, and 50 μg/mL of streptomycin. HeLa wild type (WT) and Nrf2 knockout (Nrf2^-/-^) cell lines, acquired from Abclonal (#RM01812, Wuhan, China), were cultured at a temperature of 37 °C in an atmosphere containing 5% CO_2_.

### Measurement of cell viability

Cell viability was assessed using either 3-[4,5-dimethylthiazol-2-yl]-2,5 diphenyl tetrazolium bromide (MTT) reagent (Sigma, Shanghai, China) or Cell Counting Kit-8 (CCK-8) kits (GlpBio, USA). These assays reflect cell death, changes in metabolic function, and cell proliferation. For the MTT assay, cells were incubated with 5 μg/mL of MTT for 4 hours, after which the formazan product was dissolved in 100 μL of DMSO. In the CCK-8 assay, cells were exposed to 10 μL CCK-8 solution for 2 hours. The absorbance was measured at a wavelength of 570 nm for the MTT assay and 450 nm for the CCK-8 assay, utilizing a Cytation 5 Reader (BioTek, USA). The percentage of cell viability was calculated using the formula (Optical Density of treated samples / Optical Density of control samples) × 100%. All experiments were performed in triplicate.

### Screening of ferroptosis inhibitors

In this study, we conducted a comprehensive screening of ferroptosis inhibitors using a proprietary library of 96 compounds selected for their potential relevance to ferroptosis pathways and pharmacological properties. The screening utilized a PC12 cell model with RSL-3 as the ferroptosis inducer. Each compound was tested at a concentration of 10 μM to identify those capable of rescuing cells from RSL-3-induced ferroptosis. To enhance the reproducibility and transparency of our findings, we have provided detailed descriptions of the screening protocol, including cell model preparation, assay conditions, and the chemical structures of the screened compounds, including ThA, in the Supporting Information. These additions are intended to address any concerns and strengthen the overall impact of our research, providing a valuable resource for future studies in this area.

### Lactate dehydrogenase (LDH) release assay

To assess the cytotoxic effects of ThA on PC-12 and SH-SY5Y cells, LDH release into the cell culture supernatant was measured using an LDH release assay kit (Cat. No. C0016, Beyotime Biotechnology). PC-12 and SH-SY5Y cells were cultured in six-well plates and treated with varying concentrations of ThA for 24 hours. After the treatment, the supernatant was collected and analyzed according to the manufacturer's instructions. All experiments were performed in triplicate.

### Hoechst/PI Staining

The cell death was measured using Hoechst/propidium iodide (PI) staining by identifying live cells (Hoechst-positive) and dead cells (PI-positive). Following treatment, cells were stained using Hoechst 33342 reagent to mark all nuclei and PI reagent to identify dead cells. Then, images were obtained using a fluorescence microscope. The proportion of dead cells (indicated by PI staining) to the total cell population (marked by Hoechst 33342 staining) was quantified with the help of ImageJ software (NIH, USA).

### Intracellular BODIPY 581/591 C11 staining

Lipid peroxidation within cells was determined using the BODIPY 581/591 C11 probe (Cayman, MI, USA). The cells were plated at a density of 2x10^4^ cells/well in 96-well plates. Following treatment, the cells were incubated with 50 μL of Hank's Balanced Salt Solution (HBSS) containing 40 nM BODIPY 581/591 C11 for 30 min at 37°C. Fluorescence microscopy images were obtained using a Nikon ECLIPSE 80i microscope. The ratio of green fluorescent protein (GFP) green to red fluorescence protein (RFP), which reflects the oxidation of BODIPY 581/591 C11, was analyzed and quantified using the ImageJ software.

### Intracellular ROS Assay

After treatment, cells were rinsed twice with phosphate-buffered saline (PBS) and then incubated with 10 µM dichlorodihydrofluorescein diacetate (DCFDA, #C6827, ThermoFisher Scientific, MA, USA) in HBSS for 30 min at 37 °C, away from light. Representative images of the cells were captured using fluorescence microscopy, specifically the Nikon ECLIPSE 80i model (Tokyo, Japan). The fluorescence intensity, which reflects the levels of ROS within cells, was quantified utilizing ImageJ software (NIH, USA).

### Total iron binding capacity assay

The iron chelating ability of ThA was assessed using the Total Iron Binding Capacity (TIBC) Assay Kit (A040-1-1) from the Jiancheng Institute of Biotechnology (Nanjing, China). Briefly, 1 mL of each solution—8 μM ThA, 20 μM DFO, or 4 mM FeSO_4_, all diluted in ultrapure water—was mixed with 1 mL of a 179.1 μM iron standard solution. These mixtures were allowed to stand for 10 min to facilitate interaction between the compounds and iron. Next, 50 mg of iron adsorbent was added to each mixture, thoroughly mixed, and allowed to stand for 5 min at room temperature. This process was repeated two additional times, making three cycles in total. After the final cycle, the mixture was centrifuged at 3500 rpm for 19 min. Then, 1 mL of the supernatant was carefully collected. Separate preparations included a blank group (1 mL of ultrapure water) and a standard group (1 mL of 17.91 μM iron standard solution). After adding 2 mL of iron-revealing agents to each group, the mixtures were heated in a metal bath for 5 min and then cooled to room temperature. The mixture was centrifuged at 3500 rpm for 10 min to clarify the solution further. The absorbance of 1.5 mL of the final supernatant was measured at 520 nm using a spectrophotometer. The amount of unbound iron was calculated using the formula: Unbound Iron = [(A_measurement - A_blank) / (A_standard - A_blank)] × C × 2, where C is 17.91 μM of iron standard solution.

### Isothermal Titration Calorimetry (ITC) measurement

All ITC experiments were conducted at 25°C using a Malvern MicroCal PEAQ-ITC instrument. ThA and FeSO₄ solutions were prepared in 10 mM HEPES buffer (pH 7.4) (24606001, Solarbio). The titration involved 26 consecutive injections of 1.5 μL Fe²⁺ solution into the ThA solution with continuous mixing. The reference power of the ITC cell was set to 5 cal/s, and the measurement interval between injections was 150 seconds. To account for heat changes unrelated to binding, the heat of dilution was subtracted from the raw data. Thermodynamic parameters, including dissociation constant binding enthalpy (Δ*H*), and Gibbs Free Energy change (Δ*G*) 18, were determined by fitting the binding isotherms to a single-site binding model using the MicroCal PEAQ-ITC analysis software.

### UV-visible absorbance measurements

UV-visible absorbance measurements were conducted at room temperature using an Agilent 8453 single-beam UV-visible spectrophotometer. Absorbance spectra were recorded over the 200-800 nm range using a 1 mL quartz cuvette with a 1 cm path length. A 5 mM stock solution of ThA was prepared in DMSO, and the Fe²⁺ binding properties of ThA were assessed in PBS buffer (10 mM, pH 7.4). Increasing concentrations of Fe²⁺ (0, 2, 4, 6, 8, 10, 20, 40 mM) were sequentially added to the ThA solution. After each addition, the mixture was incubated for 2 min and centrifuged to obtain the supernatant. Changes in the absorbance spectra were recorded after each titration. The data were analyzed using OriginPro 2022 software, and the absorbance values were fitted using the Benesi-Hildebrand equation to evaluate the interaction between ThA and Fe²⁺ 24, 25.

### PGSK staining

The levels of cytoplasmic iron in PC-12 cells were evaluated using the PhenGreen SK (PGSK) diacetate fluorescent indicator (Cayman, MI, USA). PGSK fluorescence is quenched upon interaction with Fe^2+^. Cells were plated at a density of 2 × 10^5^ cells on 35 mm^2^ dishes and underwent treatment for predetermined periods. Subsequently, they were incubated with 10 μM PGSK for 30 min at 37 °C, rinsed with PBS, and then fixed with 4% paraformaldehyde 26. Fluorescence images were obtained and subsequently analyzed using ImageJ software.

### Mitochondrial membrane potential detection

The mitochondrial membrane potential (MMP) was evaluated using the JC-1 kit (Beyotime, C2006). Cells were plated in 96-well plates at a density of 4×10^3^ cells/well and incubated for 24 hours. Following treatment, the cells were rinsed with PBS and then stained with the JC-1 working solution for 20 min at 37°C. After staining, the wells were washed and refilled with 100 μL of culture medium. Fluorescence microscopy was used to capture images of the cells, and ImageJ software was utilized to analyze the merged red (indicative of normal cells) and green (indicative of MMP loss) fluorescence signals.

### Western blot analysis

Protein expression was evaluated by Western blots, following the methodology outlined in the literature 27. In brief, cells were lysed in 1× RIPA buffer containing Tris-HCl, NaCl, EDTA, Triton X-100, sodium dodecyl sulfate (SDS), sodium deoxycholate, and phosphatase and protease inhibitors (Topscience, Shanghai, China). This process lasted 30 min at 4 °C, succeeded by centrifugation at 12,000g for 15 min. Protein concentration was subsequently assessed using a BCA kit (Epizyme, MA, USA). The proteins were then separated on SDS-PAGE gels and transferred to polyvinylidene fluoride membranes. These membranes were blocked using PBST containing 5% milk for one hour, incubated overnight at 4 °C with primary antibodies, and later incubated with HRP-conjugated secondary antibodies. The protein bands were visualized using an ECL substrate (4A Biotech, Beijing, China) and documented using the ChemiDoc MP System (Bio-Rad, CA, USA). Quantification of the bands was conducted using ImageJ software.

### Cell transfection

PC-12 cells were cultured in either 6-well or 96-well plates until they reached 70-80% confluence. At this point, the cells were transfected with plasmids (shGPX4, p-CAX APP Swe/Ind, or pRK5-EGFP-Tau P301L) by employing Exfect 3000 Transfection Reagent (Vazyme, Nanjing, China), adhering to the guidelines provided by the manufacturer.

### Bio-Layer Interferometry analysis

The binding affinity of ThA to GPX4 was assessed using Bio-Layer Interferometry (BLI) analysis, performed on a ForteBio Octet Red96e instrument. A 96-well plate was prepared with PBS containing 1% DMSO, ThA solutions at concentrations of 200 μM, 100 μM, 50 μM, and 25 μM, and biotinylated GPX4. The biosensor tips were coated with GPX4, and a baseline measurement was established in PBS with 1% DMSO. The process of ThA binding to and dissociating from GPX4 was continuously monitored. The resulting curves were analyzed using ForteBio's dedicated software to calculate the binding kinetics and determine the kinetic constants of the interaction.

### GPX4 enzyme activity assay

The GPX4 enzyme activity was evaluated using the GPX4 inhibitor screening assay kit (#701880, Cayman, MI, USA). This assay indirectly measures GPX4 activity through a coupled reaction with glutathione reductase. GPX4 facilitates the conversion of hydroperoxides into water, generating oxidized glutathione (GSSG) in the process. GSSG is subsequently reverted to its reduced form by glutathione reductase and NADPH. This reaction leads to the conversion of NADPH to NADP^+^, accompanied by a reduction in absorbance at 340 nm. The rate of the decrease in A340 directly correlates with the activity level of GPX4. The experiment included a control group with 100% baseline GPX4 activity, a group with 20 μM RSL-3, groups with 20 μM RSL-3 plus ThA at 4, 8, and 16 μM, and groups with ThA alone at 4, 8, and 16 μM. Following the protocol steps, reactions were incubated for 40 min before the absorbance measurement at 340 nm using a spectrophotometer.

### Cellular GPX4 activity assay

The cellular GPX4 activity assay was conducted using the Glutathione Peroxidase 4 Assay Kit from Nanjing Jiancheng Bioengineering Institute (Nanjing, China). PC-12 cells were treated with RSL-3 in the presence or absence of ThA at indicated concentrations, and post-treatment, the cells were lysed to extract proteins. The assay measured the decrease in absorbance at 340 nm, reflecting the oxidation of NADPH in the presence of glutathione and glutathione reductase, which correlates with GPX4 activity. The specific activity of GPX4 was calculated and expressed as U/mg protein. All assays were performed in triplicate.

### Molecular docking

Molecular docking was performed to investigate the interactions between ThA and the GPX4 protein using AutoDock Vina for semi-flexible docking. The chemical structure of ThA, with PubChem CID: 10328286, was acquired from the PubChem database and converted for compatibility using Open Babel software. The structure of the GPX4 protein, identified by PDB ID: 5H5Q, was sourced from the Protein Data Bank (PDB). In the docking process, cyclophosphamide was allowed to move freely (treated as flexible), whereas the GPX4 protein structure was maintained as rigid. The docking simulation targeted the entire protein surface of the GPX4 protein. The outcomes of the docking were subsequently visualized with the PyMOL software.

### GSH detection

The levels of glutathione (GSH) and its oxidized form (GSSG) were measured using the GSH and GSSG assay kit (Beyotime, S0053). PC-12 cells were seeded at a density of 5×10^5^ cells/6 cm dish and incubated for 24 hours. Following treatment, the cells were rinsed with PBS and then collected by centrifugation, discarding the supernatant. Cell lysis was achieved by three times the volume of a protein removal reagent to the cell pellet, followed by two cycles of freeze-thawing using liquid nitrogen and a water bath set at 37 ºC. The lysates were then cooled for 5 min at 4 ºC and centrifuged at 10,000g for 10 min. The clear supernatant obtained was used to measure total GSH levels. To quantify GSSG, the supernatant samples were treated with a diluted GSH clearing aid (20 μL/100 μL of the sample) and GSH clearing working solution (4 μL/100 μL of the sample), followed by incubation at 25 ºC for 60 min. The absorbance was measured at 412 nm after an additional 25 min. The concentration of GSH in the samples was determined by comparing the absorbance values to a standard curve provided with the kit. The concentration of reduced GSH was calculated by subtracting twice the amount of GSSG from the total glutathione measured: GSH = Total Glutathione - 2 × GSSG.

### Measurement of MDA content

To determine the levels of malondialdehyde 28, a marker of lipid peroxidation, the malondialdehyde assay kit (Beyotime, S0131S) was utilized. PC-12 cells were plated at a density of 5×10^5^ cells/6 cm dish and incubated for 24 hours. Following drug treatment, the cells were lysed using 100 μL of protein lysis buffer. The lysates were centrifuged at 10,000g at a temperature of 4 °C for 10 min. The protein concentration of the supernatant was measured and recorded. According to the instructions provided with the kit, the MDA detection solution was prepared and combined with the cell lysate samples. This mixture was then heated to 100 ºC for 15 min, allowed to cool, and centrifuged at 1000g for 10 min. A volume of 200 μL of the supernatant was pipetted into a 96-well plate, and the absorbance was measured at 532 nm. The concentration of MDA was calculated based on a standard curve and normalized to the protein concentration in the samples.

For measuring MDA content in* C. elegans*, the nematodes were treated with drugs, washed three times with M9 buffer, and lysed, and then the MDA content was assessed using the same procedure as described for the cell samples.

### Immunofluorescence staining

For immunofluorescence staining, PC-12 cells were cultured on confocal petri dishes. Following the treatment, the cells were rinsed three times with PBS and then fixed with 4% PFA for 15 min. Subsequently, the cells were permeabilized with 0.5% Triton X-100 for 15 min at room temperature, followed by a rinse with PBS. The cells were then blocked with FBS for 1 hour and incubated with primary antibodies at 4 °C overnight. After washing with PBS containing Tween 20 (PBST), the cells were incubated with the appropriate fluorescent secondary antibody in the dark for 1 hour. Following another wash with PBST, the slides were mounted with a mounting medium, and the images were captured using a laser confocal microscope (Zeiss-LSM-980, Germany).

### RNA extraction and quantitative real-time polymerase chain reaction (qRT-PCR)

Total RNA was isolated from PC-12 cells using TRIzol reagent (Thermo Fisher Scientific, USA) following the manufacturer's instructions. Reverse transcription of RNA into cDNA was conducted using the Prime Script RT reagent kit (Trans Serum, Beijing, China) according to the manufacturer's protocol. qRT-PCR was subsequently performed to assess relative gene expression, with GAPDH as the internal control. The primer sequences used were as follows: NRF2, forward: GGTTGCCCACATTCCCAAAC, reverse: CAGGGCAAGCGACTGAAATG; GPX4, forward: CATTCCCGAGCCTTTCAACC, reverse: CACACGCAACCCCTGTACTT; GAPDH, forward: GCATCTTCTTGTGCAGTGCC, reverse: TACGGCCAAATCCGTTCACA. All experiments were conducted in triplicate.

### Measurement of cellular ATP levels

PC12 cells were cultured in 6-well plates until they reached an appropriate density. The cells were treated with RSL-3 in the presence or absence of ThA at a concentration of 8 μM. After 24 hours of treatment, cellular ATP levels were measured using the ATP Content Assay Kit (Solarbio® BC0300), following the manufacturer's instructions. All experiments were conducted in triplicate.

### Measurement of mitochondrial ATP levels

PC-12 cells were transiently transfected with the pCMV-Mito-AT1.03 plasmid, which encodes a mitochondrial-targeted ATP sensor. After 24 hours of incubation, cells were treated with RSL-3 in the presence or absence of ThA for 24 h. Then, fluorescence imaging was performed to visualize mitochondrial ATP levels. The fluorescence intensity of the sensor was then quantified using ImageJ software, providing a measure of mitochondrial ATP production.

### Maintenance of *C. elegans*

The *C. elegans* strains used in this study were obtained from the Caenorhabditis Genetics Center (CGC), with detailed information available on wormbase.org. These strains were cultivated at 20℃ on nematode growth medium (NGM) agar plates, supplemented with live *Escherichia coli* OP50 bacteria to serve as a food source. The strains used in this study included CL4176, BR5270, BR5271, CF1553, and CL2166.

### *C. elegans* paralysis

The *C. elegans* strain CL4176, engineered to express the human Aβ1-42 in its muscle cells specifically, was utilized to evaluate paralysis in the worms, employing methods previously described in the literature 29. Synchronization of CL4176 worms was achieved, and they were cultured at 16 ºC on NGM plates, with treatments either including ThA at 20 μM or excluding it. Lip-1 at 200 μM was used as a positive control. The temperature was then increased to 25 ºC to induce and sustain Aβ1-42 expression for 24 hours. Paralysis, characterized by minimal movement of the head or a complete lack of response to tactile stimulation, was monitored until all observed worms became paralyzed. These assays were performed in triplicate across three separate experimental runs.

### Food-sensing behavior assay

The assay for detecting food-sensing behavior was conducted on the *C. elegans* strains BR5270 and its control, BR5271. *Escherichia coli* OP50 bacteria were spread on plates designed with an 8 cm outer diameter and 1 cm inner diameter void of bacteria. Following a 48-hour exposure to ThA at 20 μM and Lip-1 at 200 μM, the worms were washed and placed on NGM plates, either supplemented with or devoid of *Escherichia coli* OP50 as a food source. The movement of the worms was recorded at 20-second intervals, beginning 5 min after their transfer to the plates. The rate of deceleration in their movement was calculated using the formula: (Number of body bends without food - Number of body bends with food) / Number of body bends without food, where "Number of body bends" refers to the count of body bends. The assays included at least 20 worms per group and were repeated three times to ensure reliability.

### DHE Staining

Dihydroethidium staining was employed to assess the levels of endogenous ROS triggered by Aβ or Tau proteins in the *C. elegans* strains CL4176 and BR5270 30. For this procedure, the nematodes were washed three times with M9 buffer to remove *Escherichia coli*. They were then incubated in the dark with M9 buffer containing 2 μM DHE for 60 min. Following the incubation, the worms were rewashed and placed on a 2% agarose pad on a microscope slide, immobilized with 10 mM sodium azide. Fluorescent images of 20 nematodes were captured, and the levels of ROS were quantified using Image J based on the intensity of the fluorescence observed.

### Calcein-AM staining

Iron accumulation in nematodes was assessed using a method previously outlined in the literature 31. Briefly, worms from different culture conditions were treated with 0.05 μg/mL of Calcein-AM (Glpbio, GC34061) in M9 buffer for one hour. Following the incubation, worms were washed three times with M9 buffer and positioned on a 2% agarose pad on a slide, where they were immobilized using 10 mM sodium azide. Fluorescent images of 20 nematodes were then captured and analyzed with ImageJ software. An increase in fluorescence brightness was indicative of decreased iron levels in the nematodes.

### BODIPY 581/591 C11 analysis in *C. elegans*

In the *C. elegans* strains CL4176 and BR5270 strains, the levels of lipid peroxides were evaluated using C11 BODIPY 581/591 following exposure to ThA at 20 μM and Lip-1 at 200 μM. The worms were washed three times with M9 buffer to eliminate any remaining *Escherichia coli* and then incubated in the dark with M9 buffer containing 2 μL of 40 nM C11 BODIPY 581/591 for 60 min. After the incubation, the worms were washed again and placed on a 2% agarose pad on a slide, where they were immobilized using 10 mM sodium azide. Fluorescent images of 20 nematodes were taken, and the levels of lipid peroxides were quantified based on the GFP/RFP ratio.

### RNAi bacterial feeding

To investigate the functional roles of AMPK and Nrf2 in specific genes within *C. elegans*, RNA interference (RNAi) was utilized by feeding the nematodes with bacteria engineered to express double-stranded RNA (dsRNA) targeting each respective gene. Synchronized nematodes from the CL4176 and BR5270 strains were placed onto plates seeded with bacteria expressing RNAi against the *aak-2* and *skn-1* genes. This allowed the nematodes to grow to the necessary developmental stage or age for further experiments. As a control, nematodes from both the BR5271 and CL4176 strains were grown on NGM agar plates inoculated with HT115 bacteria, which served as the non-RNAi feeding control group. Employing this RNAi feeding strategy facilitates the study of the effects of gene-specific knockdown on the physiology and development of* C. elegans*, offering valuable insights into the molecular mechanisms that underpin their roles in disease processes and responses to stress.

### Quantification of GST-4:GFP and SOD-3:GFP Expression

In the *C. elegans* strains CF1553 and CL2166, SOD-3::GFP and gst-4::GFP expression levels were quantified. Fourth-stage larvae were cultured on NGM plates supplemented with *Escherichia coli* OP50. Ferroptosis was induced using diethyl maleate at a concentration of 1 mM, followed by treatment with ThA at 20 μM and Lip-1 at 200 μM. The nematodes were then transferred onto a 2% agarose pad using platinum wires and immobilized with 10 mM sodium azide. The levels of GFP expression were quantified using ImageJ software, with analysis conducted on at least 20 worms per experimental group.

### Toxicity assay in *C. elegans*

Synchronized *C. elegans* strains at the same developmental stage were placed on NGM plates containing varying concentrations of ThA (0, 10, 40, 160, 640, and 2560 μM) along with OP50 as a food source. After 48 hours of exposure, the worms were observed and photographed under a microscope to assess any toxic effects.

### Body length measurement in *C. elegans*

Synchronized N2 *C. elegans* strains were grown until they reached the L4 stage. At this stage, nematodes were randomly selected and mounted on slides for bright-field microscopy imaging at 10x magnification. Consistent imaging conditions were maintained to ensure accurate quantitative analysis. Photographs were taken at the L4 stage and then every other day until the fifth day after the initial imaging. Body length measurements were subsequently performed using these images.

### Statistics analysis

The data are presented as the mean ± standard deviation (SD). Differences between groups were assessed using either the Student's t-test or one-way analysis of variance (ANOVA) with GraphPad Prism 8 software. For comparisons involving multiple groups, one-way ANOVA followed by Dunnett's post hoc test was employed. A *p*-value of less than 0.05 was deemed to indicate statistical significance.

## Results

### ThA inhibits ferroptosis in neuronal cells

**Figure 1:**
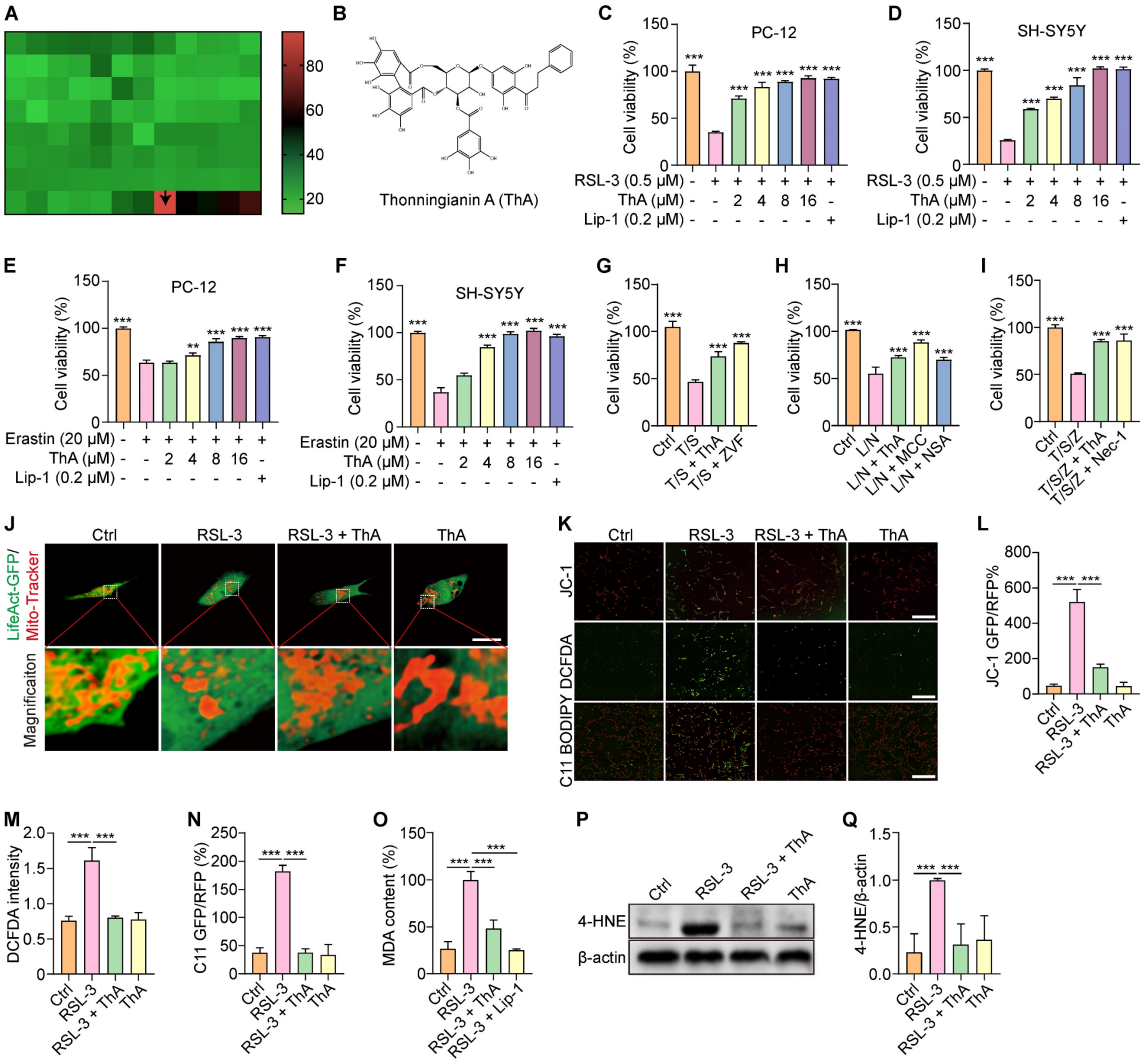
Identification of ThA as a potent inhibitor of ferroptosis. (A) Screening for inhibitors of ferroptosis in PC-12 cells exposed to RSL-3; green indicates the lowest cell viability and red represents the highest cell viability. (B) Molecular structure of ThA. (C-F) Bar charts show PC-12 and SH-SY5Y cell viability treated with 0.5 μM RSL-3 or 20 μM erastin in the presence or absence of ThA and Lip-1 at specified concentrations. (G) Bar chart indicates PC-12 cell viability treated with 10 ng/mL TNF-α and 10 nM SM-164. (T/S) with or without 8 μM ThA and 2 μM ZVF. (H) Bar chart indicates PC-12 cell viability treated with 1 μg/mL LPS and 5 μM nigericin. (L/N) in the presence or absence of 8 μM ThA, 20 μM MCC, and 10 μM NSA. (I) Bar chart indicates the viability of PC-12 cells treated with 5 ng/mL TNF-α, 1 μM SM-164, and 10 μM ZVF. (T/S/Z) with or without 8 μM ThA and 10 μM Nec-1. (J) LifeAct-GFP-transfected PC-12 cells were treated with 0.5 μM RSL-3 with or without 8 μM ThA. Mitochondrial morphology was then assessed through Mito-Tracker staining, and representative images captured. Magnification: 40×; Scale bars: 200 μm. (K) Representative images of PC-12 cells stained with JC-1-, DCFDA-, or C11 BODIPY after treatment with 0.5 μM RSL-3 with or without 8 μM ThA. Magnification: 10×; Scale bars: 200 μm. (L-N) Bar charts indicate the relative JC-1 RFP/GFP ratio, DCFDA intensity, and C11 GFP/RFP ratio. (O) Bar chart indicates the relative MDA content in PC-12 cells treated with 0.5 μM RSL-3 in the presence or absence of 8 μM ThA and 0.2 μM Lip-1. (P) Representative Western blotting images of 4-HNE and β-actin in PC-12 cells treated with 0.5 μM RSL-3 with or without 8 μM ThA. Full-length Western blot images are available in Figure S24. (Q) Bar chart indicates the ratio of 4-NHE to β-actin. Bar, SD. *, *p* < 0.05; **, *p* < 0.01; ***, *p* < 0.001.

Natural products are esteemed for their diverse chemical compositions and distinct biological activities, making them assets in discovering new therapeutic agents 32. Our study employed a library of compounds with diverse structures to find those that can inhibit ferroptosis in PC-12 cells, which were induced with RSL-3 to trigger ferroptosis (Figure S1 and Table S1). Our preliminary investigations have focused on identifying potent inhibitors from this pool, employing Fer-1 and Lip-1 as standard references due to their proven efficacy in reducing lipid peroxide accumulation in AD models. The choice to focus on Lip-1 in our study stemmed from its lower effective concentrations needed to inhibit ferroptosis than Fer-1 (Figure S2). Our findings demonstrated that ThA significantly improved cell survival (Figure 1A-B). Furthermore, ThA at 2-16 μM increased cell viability in a dose-dependent manner in both PC-12 and SH-SY5Y cells when exposed to 0.5 μM RSL-3 or 20 μM erastin (Figure S3 and Figure 1C-F). Notably, ThA effectively prevented ferroptosis induced by RSL-3 at all observed time points (4, 8, 12, and 24 hours), progressively enhancing the recovery rate of cell viability over time, thus showing both immediate and sustained protective actions against ferroptosis (Figure S4).

To explore whether ThA could also safeguard against cell death via apoptosis, pyroptosis, and necrosis, we employed specific inducers for each pathway and evaluated cell viability using the MTT assay. The results indicated that both ThA and specific inhibitors successfully restored cell viability (Figures 1G-I). This research primarily focuses on the inhibition of neuronal ferroptosis by ThA. Given the pivotal role of mitochondrial damage in initiating ferroptosis through iron-dependent oxidative stress and lipid peroxidation, we further investigated the impact of ThA on mitochondria after transiently transfecting PC-12 cells with the LifeAct-GFP plasmid and applying Mito-Tracker staining. Figure 1J showed a significant improvement in mitochondrial morphology following ThA treatment. To assess the MMP, we utilized the JC-1 kit, and our findings indicated an increase in MMP in PC-12 cells treated with ThA after RSL-3 induction (Figure 1K-L). Additionally, Figures 1K, M-Q revealed that ThA effectively reduced the production of ROS, lipid peroxides, MDA content, and 4-HNE protein expression in PC-12 cells treated with RSL-3. Our data suggested that ThA serves as an effective agent in preventing ferroptosis in neuronal cells.

### ThA inhibits ferroptosis through iron chelation

**Figure 2:**
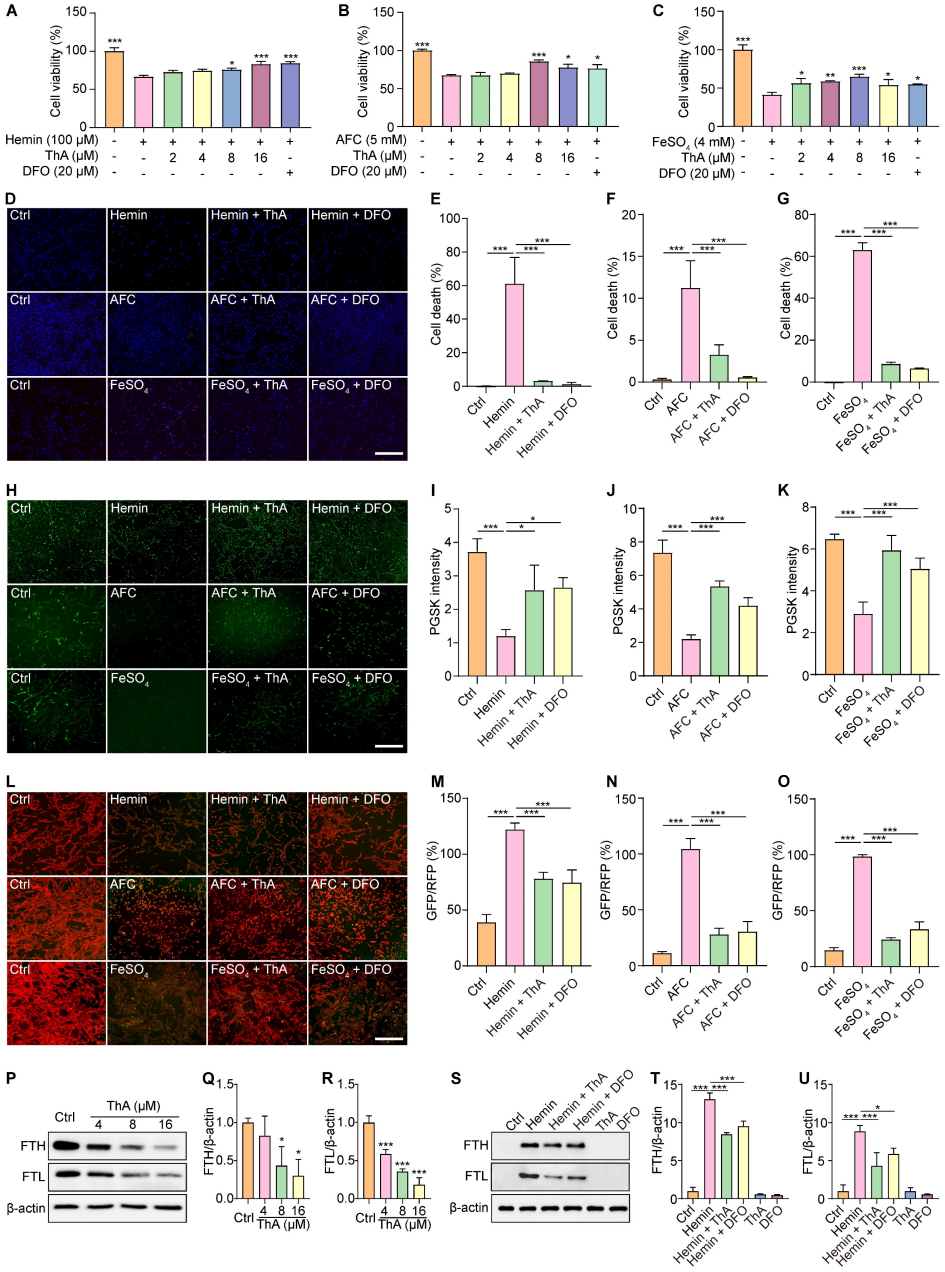
ThA chelates iron to regulate iron storage and inhibit ferroptosis. (A-C) Bar charts indicate the viability of PC-12 cells treated with 100 μM hemin, 5 mM AFC, and 4 mM FeSO_4_, with or without ThA and DFO at indicated concentrations. (D) Representative images of PC-12 cells stained with Hoechst 33342 and PI reagents after treatment with 100 μM hemin, 5 mM AFC, and 4 mM FeSO_4_ in the presence or absence of 8 μM ThA and 20 μM DFO. Magnification: 10×; Scale bars: 200 μm. (E-G) Bar charts indicate cell death in PC-12 cells under specified treatments. (H) Representative images of PC-12 cells stained with PGSK following treatment with 100 μM hemin, 5 mM AFC, and 4 mM FeSO_4_, with or without 8 μM ThA and 20 μM DFO. Magnification: 10×, Scale bars: 200 μm. (I-K) Bar charts indicate the relative PGSK intensity under specified treatments. (L) Representative images of PC-12 cells stained with C11 BODIPY after treatment with 100 μM hemin, 5 mM AFC, and 4 mM FeSO_4_ in the presence or absence of 8 μM ThA and 20 μM DFO. (M-O) Bar charts indicate the relative C11 RFP/GFP ratio. (P) Representative Western blotting images of FTH, FTL, and β-actin in PC-12 cells treated with ThA at indicated concentrations. Full-length Western blot images are displayed in Figure S25. (Q-R) Bar charts indicate the ratios of FTH and FTL to β-actin. (S) Representative Western blotting images of FTH, FTL, and β-actin in PC-12 cells treated with hemin, with or without ThA and DFO at specified concentrations. Full-length Western blot images are presented in Figure S26. (T-U) Bar charts indicate the ratio of FTH and FTL to β-actin. Bar, SD. *, *p* < 0.05; **, *p* < 0.01; ***, *p* < 0.001.* p* values determined by one-way ANOVA analysis.

Ferroptosis is an iron-dependent form of regulated cell death characterized by iron accumulation and lipid peroxidation 33. We employed three widely recognized iron inducers—hemin, AFC, and FeSO4—to induce ferroptosis in PC-12 cells and investigated whether ThA inhibits ferroptosis through its iron-chelating properties. Subsequently, we assessed the impact of ThA on cell death, iron accumulation, lipid peroxidation, and iron storage. The results showed that ThA, along with DFO (a recognized iron chelator), significantly enhanced the viability of PC-12 cells challenged with hemin, AFC, or FeSO_4_ (Figure 2A-C). Furthermore, UHPLC-DAD-Q/TOF-MS/MS analysis indicated a decrease in the intensity of both ThA and DFO in the ThA+hemin and DFO+hemin groups compared to ThA and DFO alone groups, highlighting ThA's iron-chelating capabilities (Figure S5). The total iron binding assay further demonstrated strong binding of ThA to FeSO_4_ (Figure S6). Additionally, the pronounced binding potential of ThA towards Fe^2+^ was reinforced by UV-vis absorption spectroscopy and ITC studies, wherein the *K*_D_, ΔH, and ΔG values were 1.31 μM, -79.3 kcal/mol, and -8.03 kcal/mol, respectively. These data revealed that ThA has a strong complexation with Fe^2+^ (Figure S7-8).

Further investigation using Hoechst/PI staining to detect cell death showed that ThA and DFO markedly reduced cell death in PC-12 cells triggered by hemin, AFC, or FeSO_4_ (Figure 2D-G). We used the PGSK probe to assess iron accumulation, which showed that ThA and DFO significantly increased GFP intensity in cells treated with these iron inducers, indicating reduced iron accumulation (Figure 2H-K). Employing the C11 BODIPY reagent to measure lipid peroxides in PC-12 cells revealed that ThA and DFO significantly lowered the GFP/RFP ratio, suggesting a decrease in lipid peroxidation (Figure 2L-O). The ferritin complex, comprising FTH and FTL proteins, plays a vital role in iron storage and the modulation of ferroptosis by managing iron levels and oxidative stress. Our study also examined the impact of ThA on the expression levels of FTH and FTL by Western blot analysis. The results showed that ThA reduced the expression of both FTH and FTL in a dose-dependent manner (Figure 2P-R). Notably, PC-12 cells induced with hemin exhibited significantly increased levels of FTH and FTL, while treatment with ThA or DFO effectively lowered their expression (Figure 2S-U). Our findings suggested that ThA acts as an effective iron chelator, regulating iron storage and thus inhibiting ferroptosis in PC-12 cells.

### ThA binds with GPX4 to enhance its activation and inhibit ferroptosis

**Figure 3:**
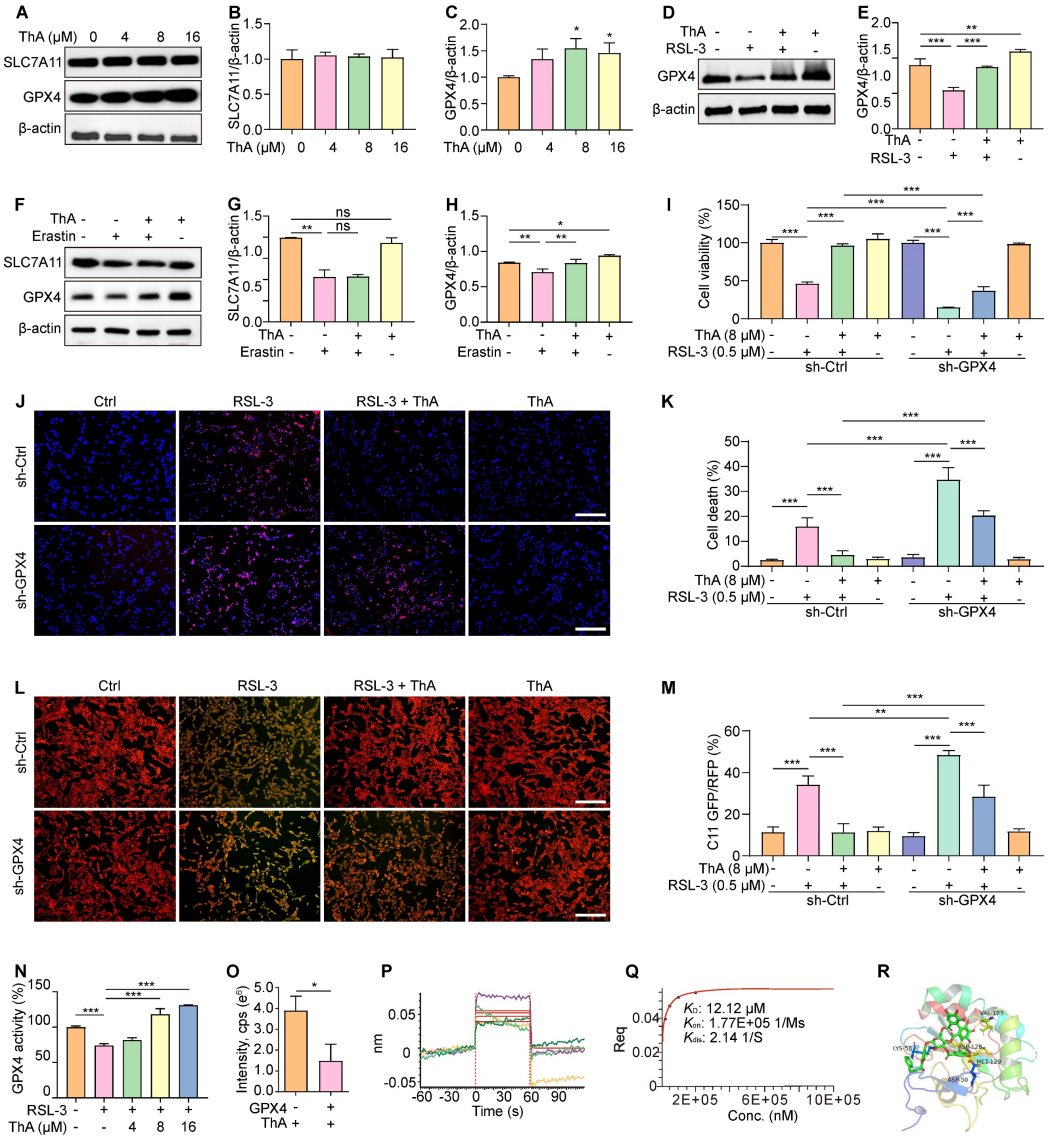
ThA interacts with GPX4 to boost its activation and inhibit ferroptosis. (A) Representative Western blot images of SLC7A11, GPX4, and β-actin in PC-12 cells treated with ThA at specified concentrations. Full-length Western blot images are presented in Figure S27. (B, C) Bar charts indicate the ratio of SLC7A11 and GPX4 to β-actin. (D) Representative Western blot images of GPX4 and β-actin in PC-12 cells treated with 8 μM ThA in the presence or absence of 0.5 μM RSL-3. Full-length Western blot images are presented in Figure S28. (E) The bar chart indicates the ratio of GPX4 to β-actin. (F) Representative Western blot images of SLC7A11, GPX4, and β-actin in PC-12 cells treated with 8 μM ThA in the presence or absence of 20 μM erastin. Full-length Western blot images are presented in Figure S29. (G, H) Bar charts indicate the ratio of SLC7A11 and GPX4 to β-actin. (I) The bar chart demonstrates the viability of sh-Ctrl or sh-GPX4 overexpressing PC-12 cells treated with 8 μM ThA in the presence or absence of 0.5 μM RSL-3. (J) Representative images of Hoechst/PI-stained sh-Ctrl or sh-GPX4-overexpressing PC-12 cells treated with 8 μM ThA in the presence or absence of 0.5 μM RSL-3. Magnification: 10×, Scale bars: 200 μm. (K) The bar chart shows cell death in Hoechst/PI-stained PC-12 cells. (L) Representative images of C11 BODIPY-stained sh-Ctrl or sh-GPX4-overexpressing PC-12 cells treated with 8 μM ThA in the presence or absence of 0.5 μM RSL-3. Magnification: 10×, Scale bars: 200 μm. (M) The bar chart indicates the GFP/RFP ratio of C11 BODIPY-stained PC-12 cells. (N) The bar chart indicates the GPX4 activity in 20 μM RSL-3 and RSL-3 with ThA at specified concentrations. (O) The chart shows the peak intensity of ThA with or without GPX4 protein, measured by UHPLC-DAD-Q/TOF-MS/MS. (P) Real-time kinetic binding sensorgrams depict different concentrations of ThA, where the response reflects the optical thickness on the SA biosensor layer. (Q) The plateau of the curve indicates the equilibrium binding signal. (R) Predicted binding modes of ThA against GPX4 protein (PDB: 5H5Q) through interactions with residues LYS-58, VAL-125, ASP-128, ASP-50, and MET-129. Bar, SD. *, *p* < 0.05; **, *p* < 0.01; ***, *p* < 0.001.

The enzyme GPX4 plays a critical role in protecting cellular membranes against damage from lipid peroxidation by preventing the accumulation of reactive lipid species and thus maintaining membrane integrity 34. Our study investigated the effect of ThA on regulating the SLC7A11/GPX4 axis through Western blotting analysis. Figures 3A-C show that GPX4 protein expression increased dose-dependently following ThA treatment, while SLC7A11 protein expression remained unchanged. ThA significantly restored GPX4 protein expression in PC-12 cells induced by RSL-3 or erastin without altering SLC7A11 protein levels (Figures 3D-H). To further understand the role of GPX4 in ThA-mediated inhibition of RSL-3-induced cell death and lipid peroxide formation, we used transient transfection with sh-Ctrl or sh-GPX4 in PC-12 cells treated with RSL-3, with or without ThA. Figures 3I-K demonstrate that ThA could not rescue PC-12 cells from RSL-3-induced death when GPX4 was knocked down, indicating that inhibition of ferroptosis by ThA was GPX4-dependent. Moreover, lipid peroxide levels were higher in RSL-3-induced PC-12 cells transfected with sh-GPX4 than sh-Ctrl, highlighting the crucial role of GPX4 in controlling lipid peroxidation (Figures 3L-M). Thus, the exacerbation of ferroptosis by RSL-3 in GPX4 knockdown PC-12 cells confirmed that ThA's protective effects depended on GPX4 activation. Furthermore, ThA independently increased GPX4 activity and dose-dependently reversed the RSL-3-induced inhibition of GPX4 activity, restoring cellular GPX4 activity in RSL-3-induced PC-12 cells (Figure 3N and S9-10).

We explored the interaction between ThA and GPX4 using UHPLC-DAD-Q/TOF-MS/MS and BLI analysis. Figure 3O indicates a decrease in ThA intensity within the ThA+GPX4 group compared to the ThA alone group, suggesting that binding of ThA to GPX4 reduces the free concentration of ThA. The BLI analysis revealed the interaction strength between ThA and GPX4, with kinetic parameters such as dissociation affinity 35. The association rate (Kon) and dissociation rate were measured as 12.12 μM, 1.77E+05 1/Ms, and 2.14 1/S, respectively (Figures 3P-Q). We conducted molecular docking using the Autodock4.2.6 software to identify the preferred binding site and theoretical binding mode of ThA on the GPX4 protein. Docking calculations determined ThA binding energy of -8.65 kcal/mol with GPX4, with significant interactions at LYS-58, ASP-128, VAL-125, MET-129, and ASP-50 residues (Figure 3R). Thus, our findings indicated that ThA interacts with GPX4, enhancing its activation and inhibiting ferroptosis in PC-12 cells induced by RSL-3 or erastin.

### ThA activates the Nrf2/Keap1 pathway

**Figure 4:**
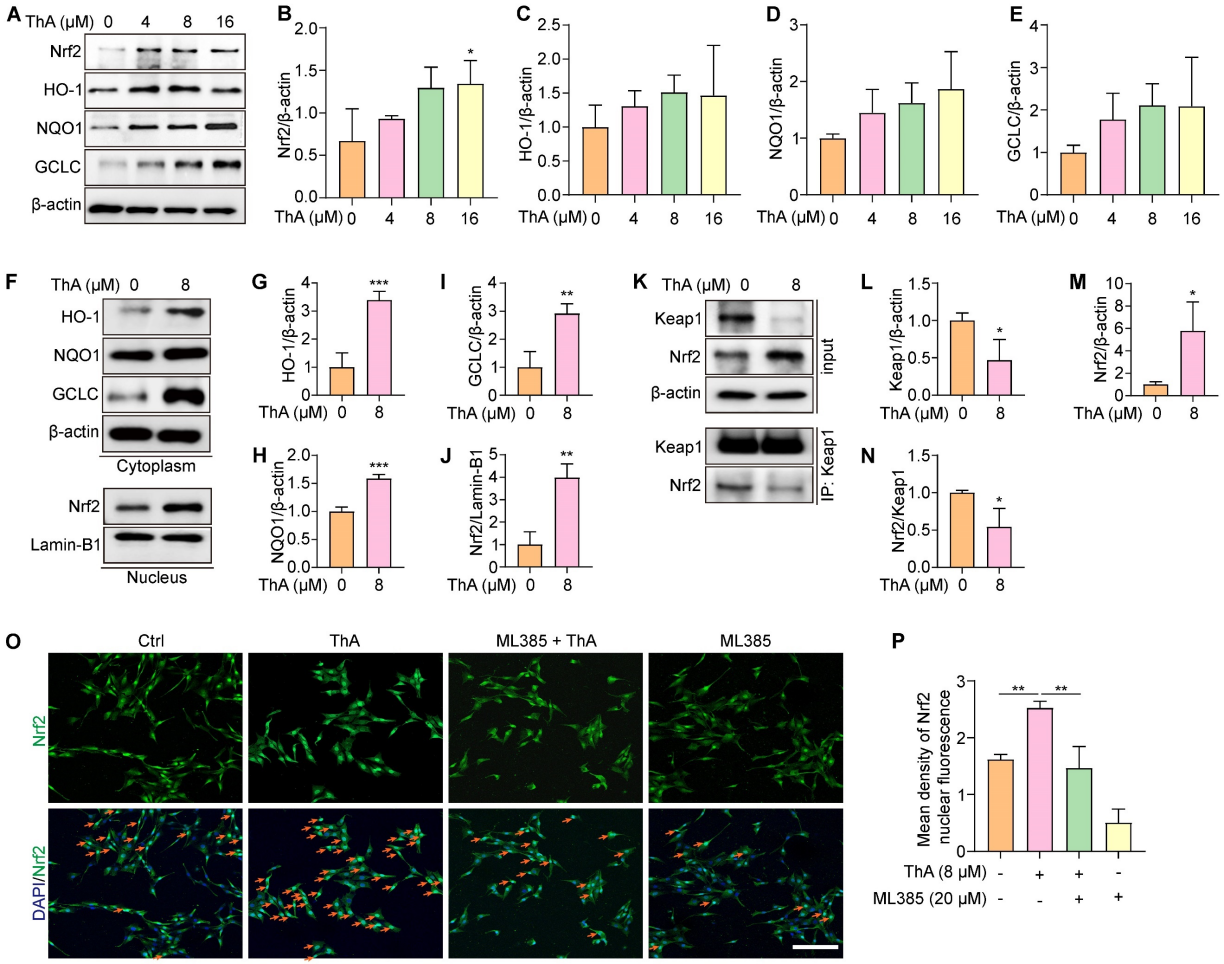
ThA activates the Nrf2/Keap1 pathway. (A) Representative Western blot images of Nrf2, HO-1, NQO1, GCLC, and β-actin in whole lysates of PC-12 cells treated with various concentrations of ThA. Full-length Western blot images are presented in Figure S30. (B-E) Bar charts indicate the ratios of Nrf2, HO-1, NQO1, and GCLC to β-actin. (F) Representative Western blot images of HO-1, NQO1, GCLC, and β-actin in the cytoplasmic lysate, and Nrf2 and Lamin-B1 in the nuclear lysate of PC-12 cells treated with 8μM ThA. Full-length Western blot images are presented in Figure S31. (G-J) Bar charts indicate the ratios of HO-1, NQO1, and GCLC to β-actin, and the ratio of Nrf2 to Lamin-B1. (K) Representative Western blotting images of Keap1, Nrf2, and β-actin, along with immunoprecipitation images of Keap1 and Nrf2 in PC-12 cells treated with 8 μM ThA. Full-length Western blot images are presented in Figure S32. (L-N) Bar charts indicate the ratios of Keap1 and Nrf2 to β-actin and the ratio of Nrf2 to Keap1. (O) Representative immunofluorescence images showing Nrf2 nuclear translocation in PC-12 cells treated with 8 μM ThA in the presence or absence of 4 μM ML385. Red arrows indicate the cells with Nrf2 nuclear translocation. Magnification: 10×, Scale bars: 200 μm. (P) Bar charts indicate the mean density of Nrf2 nuclear fluorescence. Bar, SD. *, *p* < 0.05; **, *p* < 0.01; ***, *p* < 0.001.

The Nrf2/Keap1 pathway plays a crucial role in cellular protection against ferroptosis by upregulating genes involved in antioxidant defense and iron metabolism 36. Our study evaluated the effect of ThA on activating the Nrf2/Keap1 pathway in PC-12 cells. We observed that ThA significantly enhanced Nrf2 expression and stimulated the transcriptional activity of HO-1, NQO1, and GCLC (Figure 4A-E), indicating effective activation of the NrfF2/Keap1 pathway by ThA. Further investigation into the localization of HO-1, NQO1, GCLC, and Nrf2 revealed that ThA significantly increased the cytoplasmic expression of HO-1, NQO1, and GCLC while also inducing the nuclear expression of Nrf2 (Figure 4F-J). Additionally, we explored the potential effects of ThA on the interaction between Nrf2 and Keap1 by performing immunoprecipitation. The results showed that ThA degraded Keap1, elevated Nrf2 expression, and diminished the interaction between Nrf2 and Keap1 (Figure 4K-N), suggesting that ThA disrupts the Nrf2/Keap1 interaction, potentially leading to the pathway activation. To further validate the activation of the Nrf2/Keap1 pathway, immunofluorescence experiments were conducted with ML385, a selective Nrf2 inhibitor. Figure 4O-P showed that ML385 inhibited the nuclear translocation of Nrf2 induced by ThA. Our findings demonstrated that ThA significantly activates the Nrf2/Keap1 pathway, thereby enhancing the expression of genes that provide oxidative protection.

### ThA inhibits ferroptosis by activating the Nrf2/Keap1 pathway

**Figure 5:**
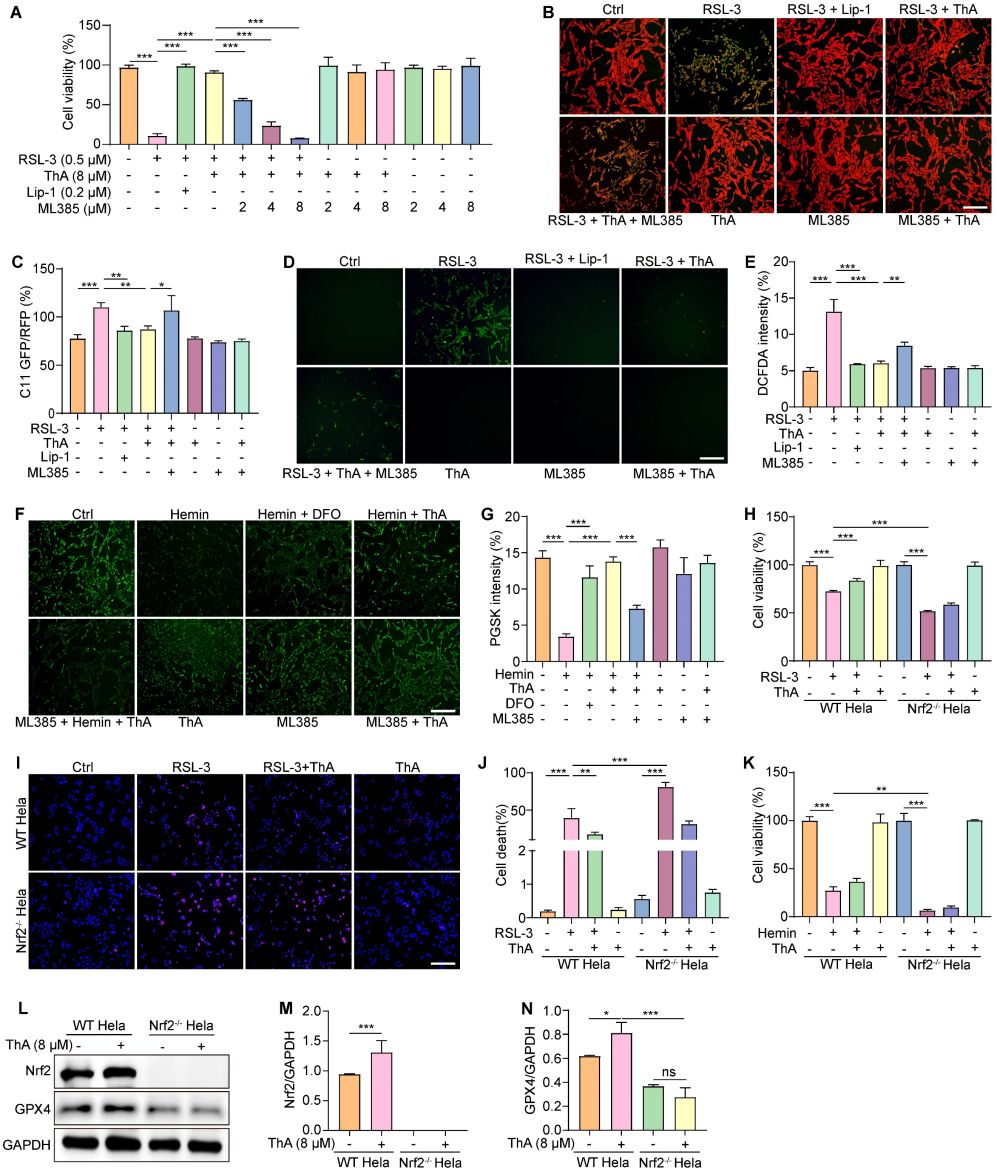
ThA inhibits ferroptosis by activating the Nrf2/Keap1 pathway. (A) Bar chartindicates PC-12 cell viability cotreated with RSL-3 and ML385 in the presence or absence of ThA and Lip-1 at specified concentrations. (B) Representative images of C11 BODIPY-stained PC-12 cells cotreated with 0.5 μM RSL-3 and 4 μM ML385 in the presence or absence of 8 μM ThA and 0.2 μM Lip-1. Magnification: 10×, Scale bars: 200 μm. (C) Bar chart indicates the C11 GFP/RFP ratio. (D) Representative images of DCFDA-stained PC-12 cells cotreated with 0.5 μM RSL-3 and 4 μM ML385 in the presence or absence of 8 μM ThA and 0.2 μM Lip-1. Magnification: 10×, Scale bars: 200 μm. (E) Bar chart indicates DCFDA intensity. (F) Representative images of PGSK-stained PC-12 cells treated with 100 μM hemin in the presence or absence of 8 μM ThA and 20 μM DFO. Magnification: 10×, Scale bars: 200 μm. (G) Bar chart indicates PGSK intensity in PC-12 cells. (H) Bar chart indicates viability of WT and Nrf2^-/-^ HeLa cells treated with 0.5 μM RSL-3 in the presence or absence of 8 μM ThA. (I) Representative images of Hoechst/PI-stained WT and Nrf2^-/-^ HeLa cells treated with 0.5 μM RSL-3 in the presence or absence of 8 μM ThA Magnification: 10×, Scale bars: 200 μm. (J) Bar chart indicates cell death of Hoechst/PI-stained HeLa cells. (K) Bar chart indicates viability of WT and Nrf2^-/-^ HeLa cells treated with 100 μM hemin in the presence or absence of 8 μM ThA. (L) Representative Western blot images of Nrf2, GPX4, and GAPDH in WT and Nrf2^-/-^ HeLa cells treated with or without 8 μM ThA. Full-length Western blot images are presented in Figure S33. (M, N) Bar charts indicate the ratios of Nrf2 and GPX4 to β-actin. Bar, SD. *, *p* < 0.05; **, *p* < 0.01; ***, *p* < 0.001.

We used ML385 to block Nrf2 activity to determine whether ferroptosis inhibition by ThA was linked to the activation of the Nrf2/Keap1 pathway. We first examined the effect of ML385 on ferroptosis inhibition by Lip-1. The results showed that Lip-1 significantly increased cell viability and inhibited lipid peroxide generation and ROS levels in RSL-3-induced PC-12 cells in the presence of ML385 (Figure S11-12). We then assessed cell viability, lipid peroxide generation, and ROS levels in PC-12 cells treated with RSL-3 with and without ThA supplementation. Figure 5A shows that ML385 effectively negated the increase in cell viability induced by ThA in RSL-3-treated PC-12 cells in a dose-dependent manner. Additionally, while ThA reduced lipid peroxide and ROS levels in RSL-3-treated PC-12 cells, co-treatment with ML385 reversed the effect of ThA (Figure 5B-E). ML385 also lessened ThA's ability to decrease intracellular iron accumulation in hemin-induced PC-12 cells (Figure 5F-G). To further validate ThA's role in inhibiting ferroptosis through Nrf2 modulation, we used WT HeLa cells and Nrf2^-/-^ HeLa cells. The results showed that RSL-3 significantly decreased the viability in Nrf2^-/-^ HeLa cells compared to WT HeLa cells (Figure S13). ThA significantly improved cell viability and reduced cell death in RSL-3-induced WT HeLa cells, but these effects were not observed in Nrf2^-/-^ HeLa cells (Figures 5H-J). Moreover, Figure 5K shows that ThA increased cell survival in hemin-induced HeLa cells but did not affect Nrf2^-/-^ HeLa cell. Western blotting revealed that ThA increased the Nrf2 and GPX4 protein expression in WT HeLa cells; however, in Nrf2^-/-^ HeLa cells, ThA failed to enhance GPX4 protein expression (Figure 5L-N). These findings suggested that ThA effectively inhibits ferroptosis by activating the Nrf2/Keap1 pathway.

### ThA inhibits ferroptosis via activating the AMPK/Nrf2/GPX4 pathway

**Figure 6:**
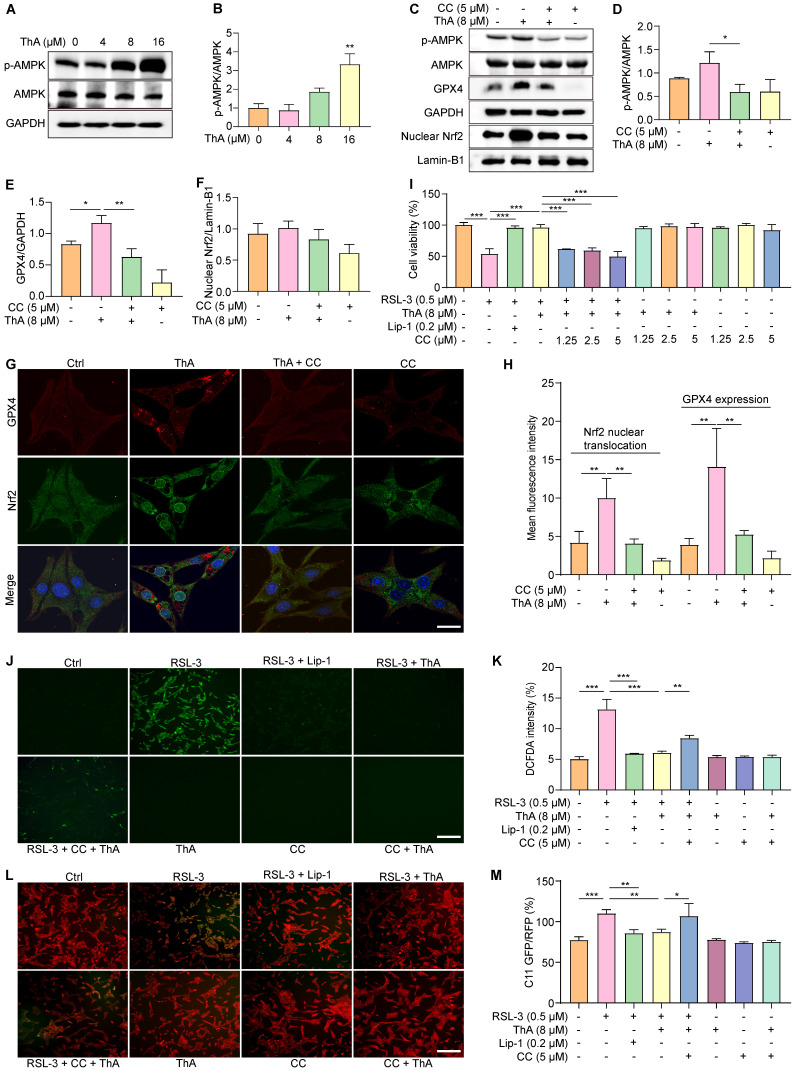
ThA inhibits ferroptosis by activating the AMPK/Nrf2/GPX4 pathway. (A) Representative Western blot images of p-AMPK, AMPK, and GAPDH in PC-12 cells treated with ThA at specified concentrations. Full-length Western blot images are presented in Figure S34. (B) Bar chart indicates p-AMPK to AMPK ratio. (C) Representative Western blot images of p-AMPK, AMPK, nuclear Nrf2, GPX4, Lamin-B1, and GAPDH in PC-12 cells treated with ThA in the presence or absence of 5 μM CC. Full-length Western blot images are presented in Figure S35. (D-F) Bar charts indicate the ratios of nuclear Nrf2 to Lamin-B1, p-AMPK to AMPK, and GPX4 to GAPDH. (G) Representative immunofluorescence images demonstrate Nrf2 nuclear translocation and GPX4 expression in PC-12 cells treated with 8 μM ThA in the presence or absence of 5 μM CC. Magnification: 63×, Scale bars: 50 μm. (H) Bar chart indicates the mean fluorescence intensity of Nrf2 nuclear translocation and GPX4 expression in PC-12 cells. (I) Bar chart indicates viability of PC-12 cells cotreated with RSL-3 and CC in the presence or absence of ThA and Lip-1 at specified concentrations. (J) Representative images of DCFDA-stained PC-12 cells treated with 0.5 μM RSL-3 and 5 μM CC in the presence or absence of 8 μM ThA and 0.2 μM Lip-1. Magnification: 10×, Scale bars: 5 μm. (K) Bar chart indicates DCFDA intensity. (L) Representative images of C11 BODIPY-stained PC-12 cells cotreated with 0.5 μM RSL-3 and 5 μM CC in the presence or absence of 8 μM ThA and 0.2 μM Lip-1. Magnification: 10×, Scale bars: 200 μm. (M) Bar chart indicates the C11 GFP/RFP ratio. Bar, SD. *, *p* < 0.05; **, *p* < 0.01; ***, *p* < 0.001.

Activation of AMPK promotes Nrf2 nuclear translocation, which, in turn, boosts the cellular antioxidant defenses and safeguards against damage induced by oxidative stress 37. Our previous research has indicated that ThA enhances autophagy in HUVEC and BV-2 cells by activating the AMPK signaling pathway 38, 39. This study aimed to explore the effect of ThA on regulating AMPK activity, leading to Nrf2 activation and ferroptosis suppression. Initially, we assessed AMPK activity by Western blotting and mRNA levels of Nrf2 and GPX4 using RT-PCR. The results demonstrated that ThA increased p-AMPK levels in a dose-dependent manner and elevated mRNA levels of Nrf2 and GPX4 in PC-12 cells (Figure 6A-B and Figure S14). However, the simultaneous application of CC negated the effect of ThA on elevating p-AMPK, GPX4, and nuclear Nrf2 protein levels (Figure 6C-F). We also used immunofluorescence to evaluate the expression and nuclear translocation and Nrf2, and the expression of GPX4. Figure 6G-H illustrates that ThA markedly promoted Nrf2 nuclear translocation and GPX4 expression, but CC treatment partially reversed these effects. Also, ML385 application effectively blocked ThA's enhancements of nuclear Nrf2 and GPX4 protein expression, Nrf2 nuclear translocation, and GPX4 fluorescence intensity (Figure S15). Furthermore, the MTT assay showed that CC dose-dependently mitigated the restorative effect of ThA on cell viability in RSL-3-induced PC-12 cells (Figure 6I). CC also partially countered ThA's reduction of ROS production (Figure 6J-K) and lipid peroxide levels (Figure 6L-M) in RSL-3-induced PC-12 cells. Our results suggested that ThA prevents ferroptosis by activating the AMPK/Nrf2/GPX4 pathway, underlining its potential as a therapeutic agent for combating ferroptosis-related conditions.

### ThA inhibits ferroptosis in cellular models of AD

**Figure 7:**
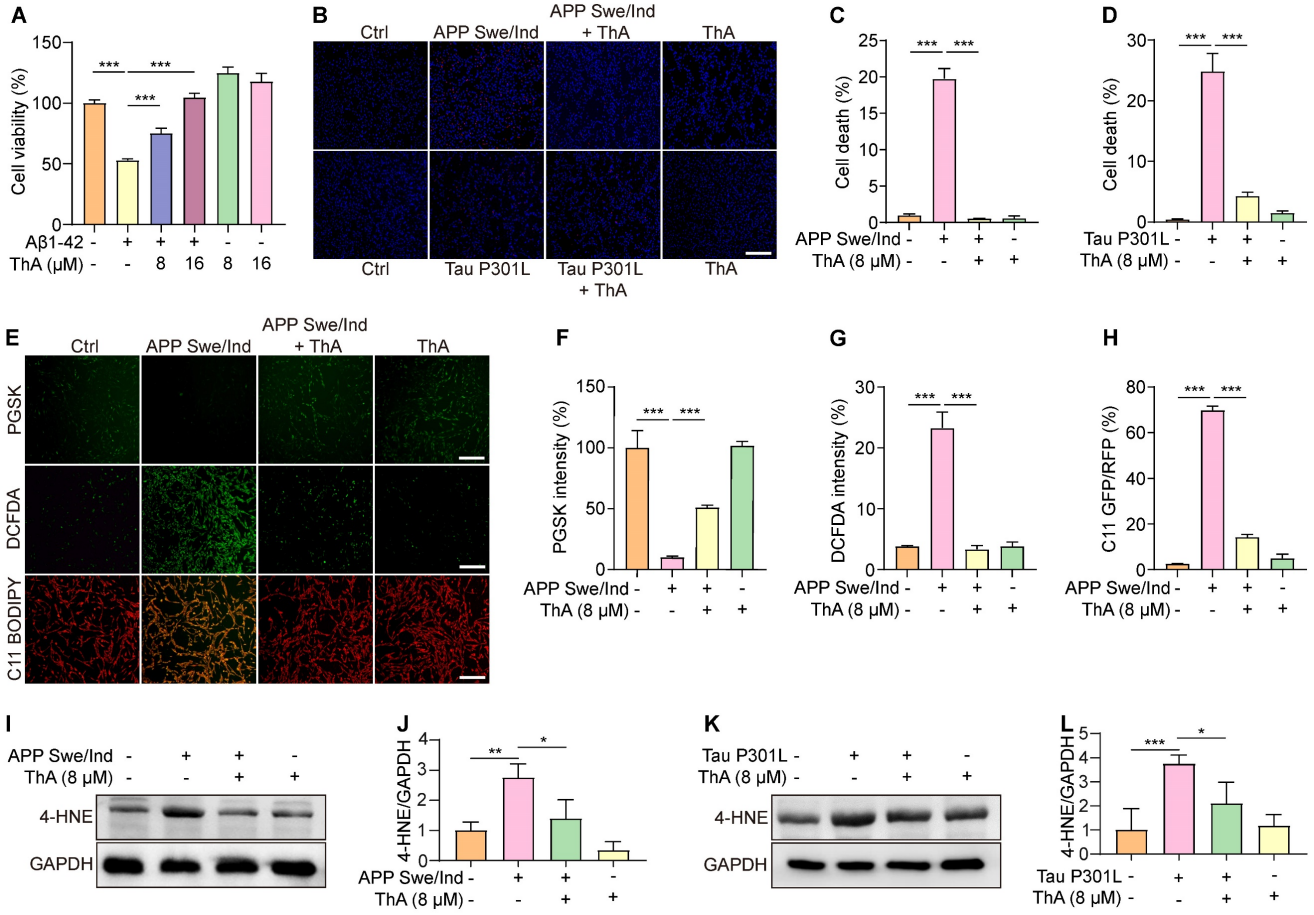
ThA inhibits ferroptosis in cellular models of AD. (A) Bar chart indicates viability of PC-12 cells treated with 10 μM Aβ1-42, with or without ThA at specified concentrations. (B) Representative images of PC-12 cells, overexpressing APP Swe/Ind or EGFP-Tau P301L, treated with 8 μM ThA and 0.2 μM Lip-1 and stained with PI and Hoechst reagents. Magnification: 10×, Scale bars: 200 μm. (C, D) Bar charts indicate PC-12 cell death. (E) Representative images of PC-12 cells overexpressing APP Swe/Ind, treated with 8 μM ThA and 0.2 μM Lip-1, and stained with PGSK, DCFDA, or C11 BODIPY reagents. Magnification: 10×, Scale bars: 200 μm. (F-H) Bar charts indicate the relative PGSK intensity, DCFDA intensity, and C11 GFP/RFP ratio. (I, K) Representative Western blot images of 4-HNE and GAPDH in PC-12 cells overexpressing APP Swe/Ind or pRK5-EGFP-Tau P301L, treated with 8 μM ThA. Full-length Western blot images are presented in Figure S36. (J, L) Bar charts indicate the ratio of 4-NHE to β-actin. Bar, SD. *, *p* < 0.05; **, *p* < 0.01; ***, *p* < 0.001.

We investigated the ThA potential in mitigating ferroptosis in AD cellular models and found that Aβ induced apoptosis, necrosis, and particularly ferroptosis in PC-12 cells (Figure S16). Subsequent analysis focused on ThA's effect on PC-12 cells exposed to Aβ1-42, revealing a significant enhancement in cell viability (Figure 7A). The APP Swe/ind plasmid was introduced into PC-12 cells and cell death was quantified using Hoechst/PI staining. As is evident from figures 7B-C, a reduction in the PI/Hoechst ratio indicated that ThA and Lip-1 decreased cell death. Similarly, ThA and Lip-1 mitigated cell death in PC-12 cells overexpressing Tau P301L (Figure 7B and D), indicating that ThA effectively protects against neuronal cell death linked to AD-related proteins.

We further explored the relationship between ThA's protective effect against cell death and its anti-ferroptotic action by assessing intracellular iron accumulation and lipid peroxidation in PC-12 cells overexpressing APP Swe/ind. PGSK staining revealed that ThA and Lip-1 significantly increased GFP intensity, indicating a reduction in intracellular iron accumulation (Figure 7E-F). Besides, DCFDA and C11 BODIPY staining demonstrated that ThA and Lip-1 lowered DCFDA intensity and the GFP/RFP ratio, suggesting decreased ROS and lipid peroxidation levels due to APP Swe/ind overexpression (Figure 7E, G, and H). Western blot analysis further confirmed that ThA substantially reduced 4-HNE expression in cells overexpressing APP Swe/ind and Tau P301L (Figures 7I-L), implying a notable decrease in lipid peroxidation levels. These results highlighted ThA's effectiveness in inhibiting ferroptosis triggered by AD-related proteins *in vitro*.

### ThA alleviates pathology and inhibits ferroptosis in* C. elegans* models of AD

**Figure 8:**
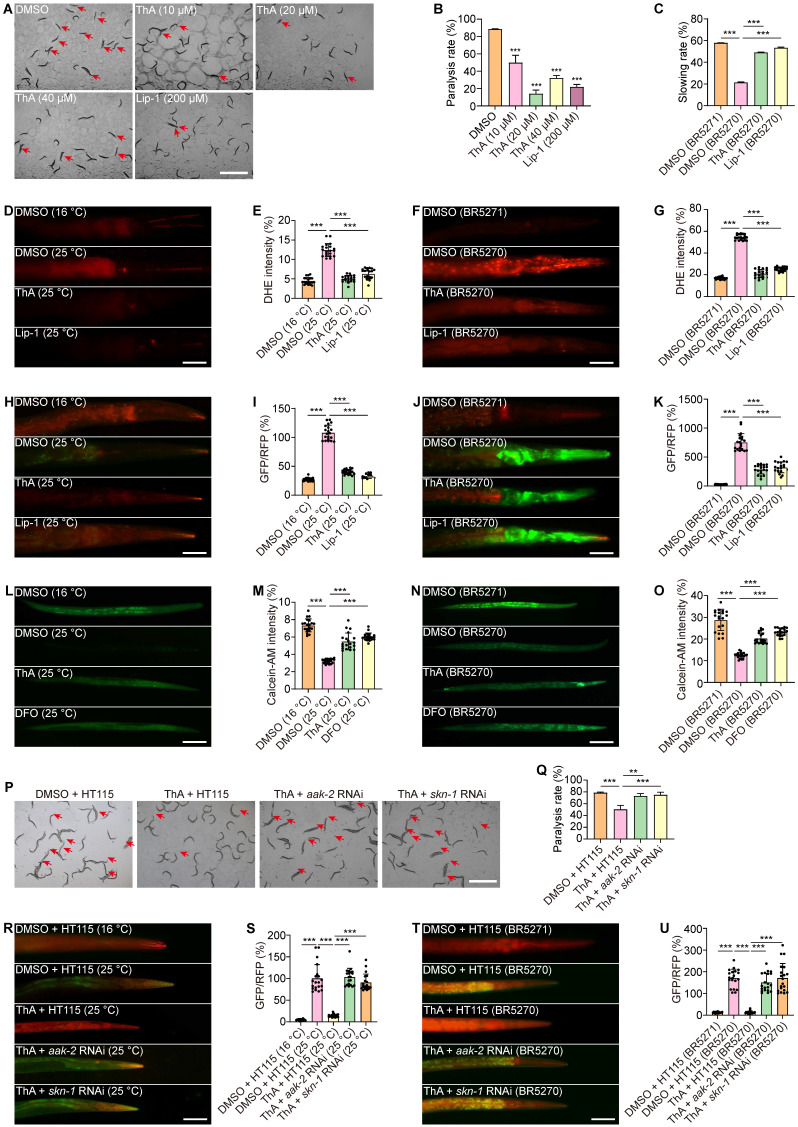
ThA inhibits ferroptosis in AD models of *C. elegans*. (A) Representative images of CL4176 worms treated with ThA and Lip-1 at specified concentrations. Red arrows highlight paralyzed worms. Magnification: ×24; scale bar: 200 μm. (B) Bar chart indicates the percentage of paralyzed worms (n = 3) with 90-100 worms counted in each group. (C) Bar chart indicates slowing rate of BR5271 and BR5270 worms treated with or without 20 μM ThA and 200 μM Lip-1 with at least 20 worms counted in each group. (D, F) Representative images of worms (CL4176, BR5271, and BR5270) stained with DHE in the presence or absence of 20 μM ThA and 200 μM Lip-1. Magnification: ×20; scale bar: 200 μm. (E, G) Bar charts indicate the relative DHE intensity in worms (n = 20). (H, J) Representative images of worms (CL4176, BR5271, and BR5270) stained with C11 BODIPY in the presence or absence of 20 μM ThA and 200 μM Lip-1. Magnification: ×20; scale bar: 200 μm. (I, K) Bar charts indicate the relative C11 GFP/RFP ratio in worms (n = 20). (L, N) Representative images of worms (CL4176, BR5271, and BR5270) stained with Calcein-AM in the presence or absence of 20 μM ThA and 200 μM Lip-1. Magnification: ×10; scale bar: 200 μm. (M, O) Bar charts indicate the relative Calcein-AM intensity in worms (n = 20). (P) Representative images of HT115 or* aak-2* RNAi and *skn-1* RNAi bacteria-fed CL4176 worms treated with or without 20 μM ThA. Red arrows indicate paralyzed worms. Magnification: ×24; scale bar: 200 μm. (Q) Bar chart indicates the percentage of paralyzed worms (n = 3), with 90-100 worms counted in each group. (R, T) Representative images of HT115 or* aak-2* RNAi and *skn-1* RNAi bacteria-fed worms (CL4176, BR5271, and BR5270) stained with C11 BODIPY in the presence or absence of 20 μM ThA. Magnification: ×20; scale bar: 200 μm. (S, U) Bar charts indicate the relative C11 GFP/RFP ratio in CL4176, BR5271, and BR5270 worms (n = 20). Bar, SD. *, *p* < 0.05; **, *p* < 0.01; ***, *p* < 0.001.

We investigated the inhibitory effects of ThA on ferroptosis in live organisms by using AD transgenic *C. elegans* strains CL4176 and BR5270. Initially, we examined the impact of ThA on the behavioral performance of the nematodes. Figure 8A-B demonstrated that ThA and Lip-1 significantly delayed the onset of paralysis in CL4176 worms, specifically expressing human Aβ1-42 in their muscles. Also, ThA and Lip-1 markedly improved the food-sensing deficits observed in BR5270 worms (Figure 8C). We further assessed ferroptosis in *C. elegans* by measuring ROS levels, lipid peroxides, and iron accumulation. DHE staining images showed that ThA and Lip-1 significantly reduced DHE fluorescence intensity in CL4176 and BR5270 strains, indicating a decrease in ROS levels in AD worms (Figures 8D-G). Moreover, Figure 8H-K revealed that ThA and Lip-1 decreased the GFP/RFP ratio, suggesting reduced lipid peroxide levels in worms stained with C11 BODIPY. Iron accumulation in these worms was also evaluated using Calcein-AM. Figures 8L-O show a significantly lowered GFP intensity by ThA and DFO, indicating reduced iron levels in the worms.

We also explored the antioxidative properties of ThA and found decreased MDA content in both CL4176 and BR5270 strains (Figure S17) and diminished expression of SOD-3 and gst-4 in CF1553 and CL2166 transgenic worms (Figure S18-19). To ascertain the role of the AMPK/Nrf2 pathway in ThA's action against ferroptosis, we conducted targeted gene knockdown experiments in CL4176 and BR5270 strainsof *C. elegans*. We specifically reduced the expression of *aak-2* (the *C. elegans* equivalent of human AMPK) and *skn-1* (equivalent to mammalian Nrf2) using RNAi bacteria. Our results indicated that knocking down* aak-2* and* skn-1* significantly impaired ThA's capacity to lower paralysis rates and lipid peroxide production in both strains (Figures 8P-U).

Finally, the biosafety and acute toxicity of ThA were evaluated in N2 worms. Treatment with ThA had a dose-dependent effect on the body length of *C. elegans* over time (Day 1, 3, and 5). Low to moderate concentrations of ThA (10-160 µM) generally increased body length compared to the control, especially on days 3 and 5, while the highest concentrations (640-2560 µM) significantly reduced body length and caused severe morphological abnormalities, indicating toxicity at this level (Figure S20). In summary, these findings suggested that ThA has relatively high biosafety, and inhibits ferroptosis in* C. elegans* models of AD primarily mediated via the activation of the AMPK/Nrf2 pathway.

## Discussion

Ferroptosis, a recently identified form of regulated cell death, is distinguished by uncontrolled iron accumulation and lipid peroxide production, setting it apart from apoptosis, necrosis, autophagy, and other cell death modalities in terms of appearance, chemical reactions, and genetic markers 40. The unique ultrastructural hallmark of ferroptosis is mitochondrial morphological changes. Comprehensive studies have shown that ferroptosis plays a significant role in various diseases, including liver injury, cancer, cardiovascular disease, and neurodegenerative disorders 9, 41-45.

There has been considerable research aimed at elucidating the links between oxidative stress related to ferroptosis, iron buildup, lipid peroxidation, mitochondrial damage, and the onset of neurodegeneration 12, 46-48. Initially, cancers were identified as a primary condition associated with ferroptosis, leading to extensive research into its occurrence in neurodegenerative diseases. Both direct and indirect evidence increasingly highlight the crucial involvement of ferroptosis in neuronal death and neuroinflammation, observed in the laboratory (*in vitro*) and living organisms (*in vivo*) 12, 49-51. In patients with neurological conditions, increased lipid peroxidation has been noted in the brain tissue and cerebrospinal fluid 52. Iron accumulation in the brain is closely linked to disease progression in individuals with cognitive decline and AD 53, 54. Furthermore, treatment with iron chelators such as deferiprone or DFO has been effective in AD clinical trials 21, 55. Ferroptosis inducers significantly promote neuronal death *in vitro*, associated with mitochondrial damage and excessive ROS production. However, treatment with ferroptosis inhibitors and the ROS scavenger N-acetylcysteine blocks cell death 56, 57. Other studies have reported that GPX4, an enzyme that converts lipid peroxides into non-toxic lipid alcohols using glutathione, prevents ferroptosis 15, 58. Emerging evidence indicates that overexpression of GPX4 inhibits lipid peroxide production and neuronal death, improving motor function in various neurodegenerative diseases such as AD 59-62. Conversely, GPX4 knockout in brain neurons exacerbates motor and cognitive dysfunction, neuronal death, and neuroinflammation in AD mice 16, suggesting that iron accumulation and associated ferroptosis contribute to AD progression and development. Despite extensive research into its pathogenesis and molecular mechanism, there is no cure for AD, prompting further investigation into its complex mechanism and discovery of therapeutically effective drugs.

In this study, we screened our established natural compound library to identify potent ferroptosis inhibitors from natural resources with high safety and minimal side effects. We identified ThA as a potent ferroptosis inhibitor in RSL-3-induced PC-12 cells and demonstrated its efficacy in cellular models of ferroptosis induced by different agents, including erastin, hemin, AFC, and FeSO_4_. ThA's ability to restore cell viability indicated a positive trend over time, providing insight into the potential mechanisms underlying its sustained protective action against ferroptosis. However, at 16 μM, ThA did not efficiently increase cell viability in AFC- or FeSO_4_-induced PC-12 cells (Figure 2B-C). Despite the absence of statistically significant differences, our observation of a slight reduction in cell viability at 16 μM of ThA alone suggests that ThA might exert prooxidant effects at this concentration 63. Future investigations will aim to systematically explore this potential mechanism to better understand the role of ThA in modulating redox states in the presence of iron. ThA, an ellagitannin derived from the African medicinal plants* Thonningia sanguinea* and *Penthorum chinense* Pursh, exhibits antioxidative, anticancer, and anti-inflammatory properties 38, 39. Recognizing the complexity of cell death pathways involved in AD, we assessed ThA's inhibitory effects across various pathways. Our findings revealed that ThA exhibits low biological toxicity and demonstrates broad-spectrum efficacy in mitigating cell death, highlighting its potential as a multi-targeting agent against AD's complex cell death mechanisms. This aspect of ThA's bioactivity broadens its therapeutic applicability and emphasizes natural compounds' potential to demonstrate diverse mechanistic actions. Furthermore, we highlight the necessity of future research to explore the molecular mechanisms of ThA across different cell death pathways, which is crucial for translating our preliminary findings into therapeutic strategies and validating in more complex *in vivo* AD models. Our previous studies have shown ThA's role in protecting against oxidative damage and mitochondrial dysfunction in human umbilical vein endothelial cells 38, emphasizing its potent antioxidative capabilities. ThA has also facilitates the degradation of the NLRP3 inflammasome via autophagy in microglial cells, offering a new approach to mitigating neuroinflammation in AD 39. These findings suggest that ThA may alleviate AD-like pathology by inhibiting neuronal ferroptosis by reducing oxidative damage and mitochondrial dysfunction. Building upon these insights, the current study has provided another dimension for ThA's therapeutic action—its capacity to inhibit ferroptosis in neuronal cells, thus broadening our understanding of ThA's protective mechanisms and its potential as a versatile therapeutic agent for neurodegenerative diseases, including AD. We further explored ThA's ferroptosis-inhibiting mechanism by examining its iron chelation and comparing its effectiveness with known iron chelators, such as DFO. ThA significantly decreased iron levels in cell-free tests, indicating a potent iron-chelating ability and its role in suppressing cellular iron accumulation and iron-dependent ferroptosis. We also investigated ThA's impact on the SLC7A11/GPX4 signaling pathway and found that ThA notably increased GPX4 protein expression independent of SLC7A11 signaling. Furthermore, using BLI 64, UHPLC-DAD-Q/TOF-MS/MS analysis, and molecular docking, we demonstrated ThA's strong binding affinity to GPX4, contributing to GPX4 activation and ferroptosis inhibition.

Nrf2 signaling is recognized for regulating ferroptosis through cellular antioxidant response, iron, lipid, and glutathione metabolism 36. Additionally, Nrf2 activation protects against oxidative, inflammatory, and proteotoxic stress in various preclinical neurodegeneration models, including AD and MS 22, 65, 66. Our study explored ThA's influence on the Keap1/Nrf2 signaling pathway since ThA facilitated the nuclear translocation of Nrf2 and the production of transcription factors like HO-1, NQO1, and GCLC. Recent studies have highlighted the importance of the Nrf2-GPX4 axis in mitigating ferroptosis and its implications for AD. For instance, it has been demonstrated that activation of the Nrf2 pathway enhances GPX4 expression, thereby providing neuroprotection against ferroptosis in AD models 18, 19. Our findings align with this concept, as ThA was shown to upregulate GPX4 levels via the Nrf2 pathway, contributing to its protective effects against ferroptosis in both cell and worm models of AD. The results showed that ThA-induced upregulation of GPX4 partially depended on Nrf2 activation, while SLC7A11 expression remained unchanged, suggesting that ThA activates the Keap1/Nrf2/GPX4 signaling pathway. The insights gained from our research indicated that ThA's protective effects are mediated through activating the AMPK pathway. Studies have shown that AMPK promotes mitochondrial biogenesis and autophagy, processes crucial for maintaining cellular homeostasis and protecting against neuronal death in various neurodegenerative diseases 67, 68. AMPK activation can inhibit ferroptosis by modulating levels of critical proteins, including ACC and FOXO3a 69, and also enhances the production of antioxidant enzymes, such as HO-1, leading to decreased peroxide accumulation and protection against ferroptosis 70. Besides, AMPK activation promotes Nrf2 nuclear translocation, enhancing antioxidant defense and reducing lipid peroxidation while inhibiting mTORC1, which reduces iron uptake and prevents the accumulation of lipid peroxides 70, 71. Our study further explored whether ThA-induced AMPK phosphorylation could promote Nrf2 nuclear translocation and GPX4 upregulation. The results indicated that CC significantly inhibited Nrf2 expression and nuclear translocation. Moreover, ML385 treatment counteracted the inhibitory effect of ThA on RSL-3-induced ferroptosis, suggesting that ThA inhibits ferroptosis via the AMPK/Nrf2/GPX4 pathway. Also, AMPK enhances ATP production and mitochondrial metabolism by stimulating energy-generating processes, such as fatty acid oxidation and glucose uptake, while also promoting mitochondrial biogenesis and efficiency to meet cellular energy demands 72. Here, we examined the effect of ThA on ATP production in RSL-3-induced PC-12 cells and found that ThA significantly restored ATP levels in RSL-3-induced PC-12 cells (Figure S21), and OA, a mitochondrial ATP synthase inhibitor, abolished the protective effect of ThA against RSL-3-induced cell death (Figure S22). These data suggested that ThA's inhibition of ferroptosis might involve mitochondrial metabolism, which requires further research.

Our previous studies demonstrated that ThA inhibits oxidative stress and mitochondrial dysfunction via autophagy induction 38. Considering our findings that ThA inhibits ferroptosis, potentially through autophagy activation, we further investigated this mechanism using autophagy inhibitors, including 3-MA and Baf. Our preliminary observations suggested that the inhibition of autophagy with 3-MA and Baf partially reversed ThA's effects on ferroptosis, indicating that autophagy may contribute to ThA's mechanism of action (Figure S23). This observation is consistent with the literature and establishes a complex relationship between autophagy and ferroptosis, where autophagy can promote and inhibit ferroptosis under different conditions. These findings should be considered in the context of existing literature and acknowledge the limitations of our experimental approach. The dual role of autophagy in cell survival and death presents a complex landscape where modulating autophagy with therapeutic agents can have protective and detrimental effects on ferroptosis, depending on the cellular context and stress stimuli. Therefore, while our findings are inconclusive, they open avenues for further in-depth investigation into the relationship between ThA-induced autophagy and ferroptosis suppression. Future studies aimed at identifying specific molecular mechanisms and signaling pathways will be crucial in establishing ThA's therapeutic potential in conditions where ferroptosis is pivotal, clarifying autophagy's dual role in ferroptosis and identifying new therapeutic targets for diseases characterized by dysregulated iron metabolism and oxidative stress.

In this study, we used APP Swe/ind or Tau P301L-overexpressing PC-12 cells to investigate ThA's ability to inhibit ferroptosis in AD cellular models. The results showed that ThA significantly inhibited APP Swe/ind- or Tau P301L-induced cell death and reduced ROS levels and lipid peroxide generation. We selected* C. elegans* as our model organism for its advantages in screening of potential therapeutic compounds targeting ferroptosis in AD. This choice was informed by our prior work, which demonstrated ThA's efficacy in improving cognitive functions and activating the AMPK pathway in AD mouse models 39. These initial findings provided compelling evidence of ThA's potential in modulating crucial pathways implicated in AD, laying a strong foundation for our subsequent exploration using *C. elegans*. The rationale for employing *C. elegans* as a model organism in this study was further supported by several key considerations.* C. elegans* shares significant genetic homology with humans, including genes involved in ferroptosis, neurodegenerative diseases, and stress response pathways 73. Its relatively simple and well-characterized nervous system allows for basic observations of neurodegenerative processes. Moreover, *C. elegans* is amenable to genetic manipulation, facilitating precise control over gene expression and creating specific disease models. Also, *C. elegans* is more cost-effective and time-efficient than mammalian models, allowing for high-throughput screening of potential therapeutic compounds like ThA. Other advantages of utilizing* C. elegans* include its short lifespan, which facilitates rapid observation of disease progression and therapeutic effects of ThA, high reproducibility due to the ease of maintaining large populations under controlled conditions, and fewer ethical concerns compared to mammalian models. Our investigations in *C. elegans* expressing Aβ or Tau provided preliminary evidence that ThA could improve behavioral performance and reduce ROS levels, lipid peroxides, iron accumulation, and oxidative stress. The attenuation of ThA's protective effects following the knockdown of *aak-2* and *skn-1* provided compelling evidence for the central role of the AMPK/Nrf2 pathway in inhibiting ferroptosis by ThA. These findings validated our *in vitro* observations and emphasized the translational potential of targeting ferroptosis pathways as a novel therapeutic strategy for AD. These data bridged a critical gap in our understanding of ThA's mechanistic action *in vivo* and provided evidence for our hypothesis that AMPK and Nrf2 activation are pivotal for ThA's anti-ferroptosis activity. Our *in vivo* studies, revealing ThA's effect via AMPK and Nrf2 pathways, reinforce the complexity of ferroptosis regulation and the need for targeted therapeutic strategies to combat neurodegenerative diseases effectively. Nevertheless, there are also disadvantages, such as the simplified physiology of *C. elegans*, which lacks some of the complex brain structures and physiological processes found in mammals, limiting the translatability of specific findings. Besides, certain aspects of AD pathology, such as amyloid plaque formation and tau pathology, are not fully recapitulated in *C. elegans*. The limitations also include the inability to accurately mimic the complexity of AD pathology, including amyloid-beta plaque formation, tau tangles, and neuroinflammation, and differences in drug metabolism and pharmacokinetics, which can affect the efficacy and safety profiles of therapeutic candidates. While *C. elegans* provides a valuable and efficient model for initial screening and mechanistic studies, validating our findings in mammalian models is imperative. Our future studies will include the use of AD mouse models to investigate the therapeutic potential of ThA and its effects on mammalian AD pathology, focusing on its ability to modulate various pathways and confer neuroprotection in more complex systems and addressing the intricacies of the disease that *C. elegans* cannot fully replicate.

In this study, our findings support ThA's potential to modulate ferroptosis, enhance neuronal viability, and improve AD markers, laying a strong foundation for future research. The focus should remain on validating ThA's efficacy in inhibiting neuronal ferroptosis in a mammalian context, with the ultimate goal of gaining meaningful insights into developing effective treatments for AD. This strategic research trajectory, from foundational studies in *C. elegans* to comprehensive validation in mammalian models, underscores our holistic approach to exploring innovative therapeutic solutions for neurodegenerative diseases. Recently, we have demonstrated that ThA could also target and inhibit AD-related Aβ and tau proteins, thereby reducing their aggregation and associated neurotoxicity 74, likely alleviating oxidative stress and lipid peroxidation, which are key contributors to ferroptosis. Therefore, the dual action of ThA in targeting Aβ/tau proteins and activating the Nrf2/GPX4 pathway affects multiple pathological processes, from oxidative stress and mitochondrial dysfunction to neuroinflammation and ferroptosis, implicated in AD underscoring its therapeutic potential in mitigating AD pathology by preventing ferroptosis and neuronal cell death. Thus, our study has identified ThA as a promising candidate for multifaceted disease intervention, underscoring the importance of integrated therapeutic strategies that can simultaneously address the diverse molecular and cellular dysfunctions characteristic of AD.

## Conclusions

**Figure 9:**
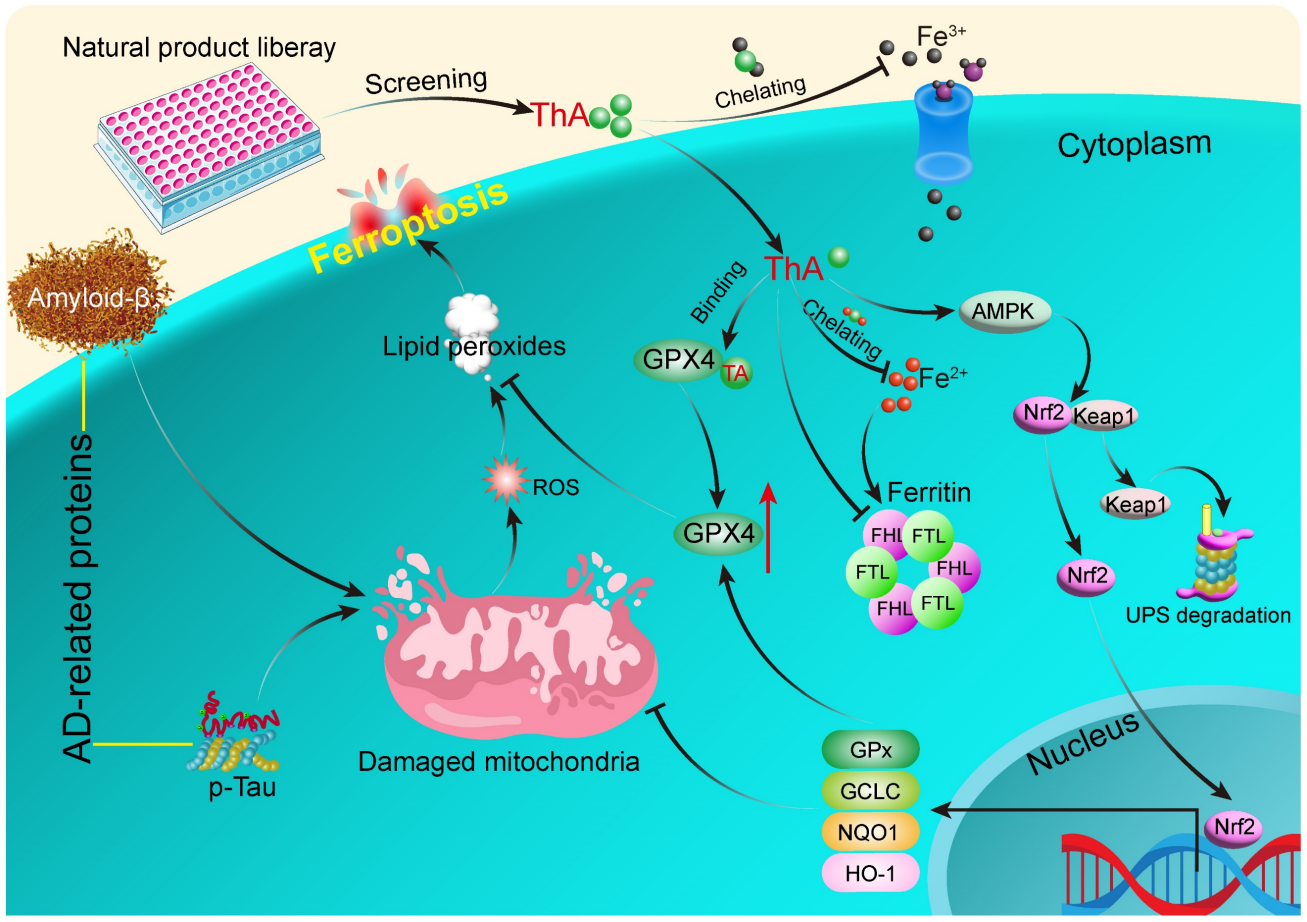
Schematic diagram of the study. ThA inhibits neuronal ferroptosis by binding with GPX4 and activating the AMPK/Nrf2/GPX4 signaling pathway to stimulate GPX4 activation. This effect has been demonstrated in cellular and *C. elegans* models of AD.

Our study provides substantial evidence that ThA, a naturally occurring compound, plays a significant role in inhibiting ferroptosis. This is achieved through its interaction with GPX4 and activating the AMPK/Nrf2/GPX4 signaling pathway, effectively blocking ferroptosis. Utilizing both cellular and *C. elegans* models of AD, we've demonstrated that ThA significantly ameliorates AD-related pathologies by counteracting neuronal ferroptosis (Figure 9). These results underscore the therapeutic potential of ThA in treating AD and the importance of further investigation into its efficacy in preclinical and clinical settings.

## Supplementary Material

Supplementary figures and table.

## Abbreviations

AD: Alzheimer's Disease
Aβ: amyloid-β
NFTs: neurofibrillary tangles
RSL-3: RAS-selective lethal compounds 3
ROS: reactive oxygen species
GPX4: glutathione peroxidase 4
Fer-1: ferrostatin-1
Lip-1: liproxstatin-1
ThA: thonningianin A
*C. elegans*: *Caenorhabditis elegans*
AMPK: AMP-activated protein kinase
Nrf2: nuclear factor erythroid 2-related factor 2
AFC: ammonium ferric citrate
NSA: necrosulfonamide
ZVF: Z-VAD-FMK
FeSO_4_.7H_2_O: iron(II) sulfate heptahydrate
OA: oligomycin A
CC: compound C
Baf: bafilomycin A1
3-MA: 3-methyladenine
Nec-1: necrostatin-1
DMEM: Dulbecco's Modified Eagle Medium
FBS: fetal bovine serum
FTH: ferritin heavy chain
FTL: ferritin light chain
GCLC: glutamate-cysteine ligase complex
p-AMPK: phosphorylated AMPK
NQO-1: NAD(P)H quinone oxidoreductase 1
4-HNE: 4-hydroxynonenal
MTT: 3-[4,5-dimethylthiazol-2-yl]-2,5 diphenyl tetrazolium bromide
CCK-8: cell counting kit-8
LDH: lactate dehydrogenase
PI: propidium iodide
GFP: green fluorescent protein
RFP: red fluorescence protein
PBS: phosphate-buffered saline
DCFDA: dichlorodihydrofluorescein diacetate
TIBC: Total Iron Binding Capacity
ITC: Isothermal Titration Calorimetry
PGSK: PhenGreen SK
MMP: Mitochondrial membrane potential
BLI: Bio-Layer Interferometry
PDB: Protein Data Bank
GSSG: generating oxidized glutathione
GSH: glutathione
MDA: malondialdehyde
qRT-PCR: real-time polymerase chain reaction
CGC: Caenorhabditis Genetics Center
NGM: nematode growth medium
DHE: dihydroethidium
RNAi: RNA interference
SD: Standard deviation
ANOVA: one-way analysis of variance
