# Supplementary material for: A novel ferroptosis inhibitor, Thonningianin A, improves Alzheimer's disease by activating GPX4
Source: Theranostics. 2024 Sep 23;14(16):6161–84. doi: 10.7150/thno.98172 (PMC11488096; doi:10.7150/thno.98172)
Supplement: Supplementary file 1 — Supplementary figures and table. [file thnov14p6161s1.pdf]

Supplementary materials for

A novel ferroptosis inhibitor, Thonningianin A, improves Alzheimer's disease by activating GPX4

Authors: Yuanyuan Yong[a,†], Lu Yan[a,†], Jing Wei[a,b,†], Chi Feng[a,†], Lu Yu[a], Jianming Wu[a], Minsong Guo[a], Dongsheng Fan[c], Chonglin Yu[a], Dalian Qin[a,*], Xiaogang Zhou[a,*], Anguo Wu[a,*]

*Corresponding author(s). Tel.: +86-17769617417; fax:+86-083031361222.

E-mail address(es): Dalian Qin, dalianqin@swmu.edu.cn; Xiaogang Zhou, zxg@swmu.edu.cn; Anguo Wu, wuanguo@swmu.edu.cn.

This Word file includes:

Figures S1 to S37 and Table S1

A1

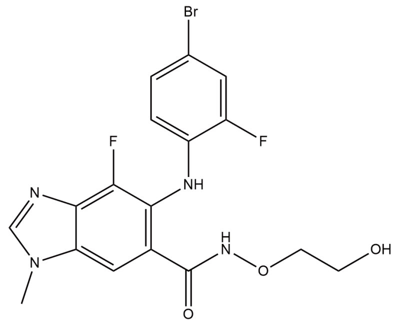

A2

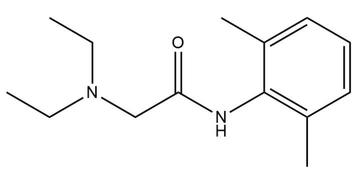

A3

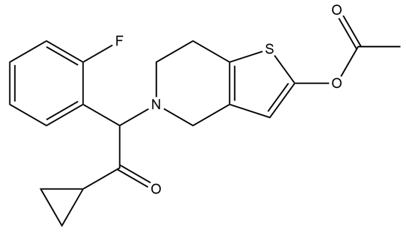

A4

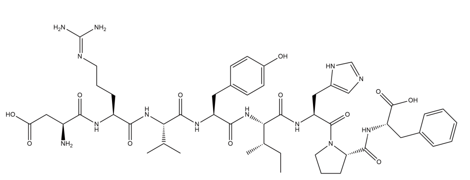

A5

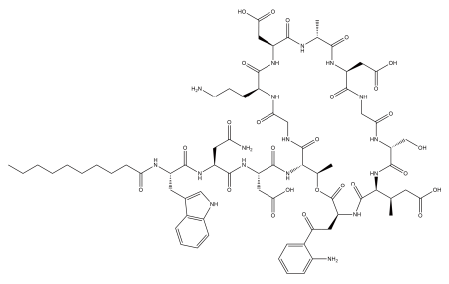

A6

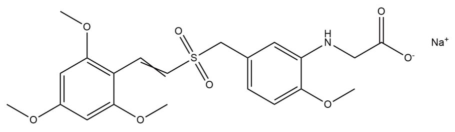

A7

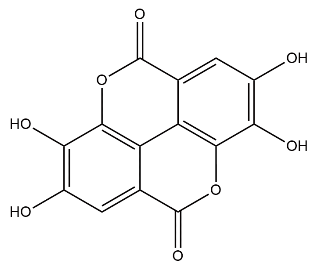

A8

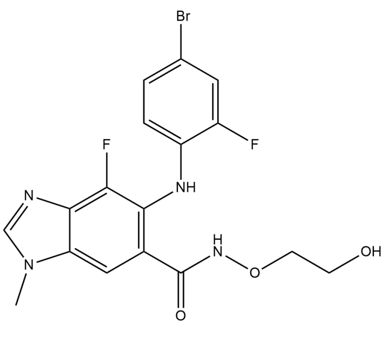

A9

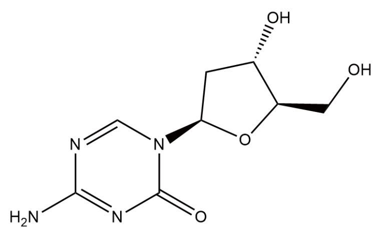

A10

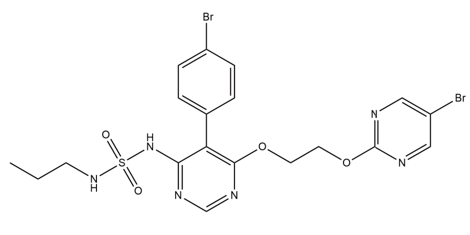

A11

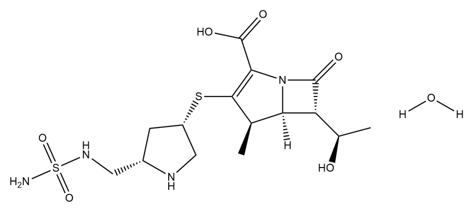

A12

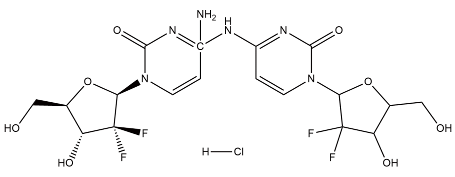

B1

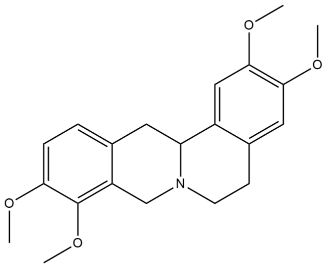

B2

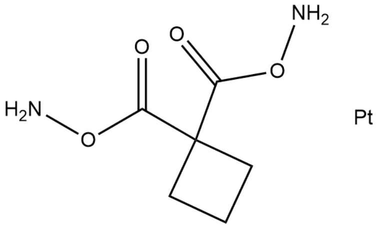

B3

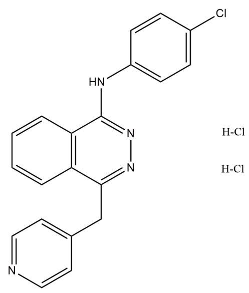

B4

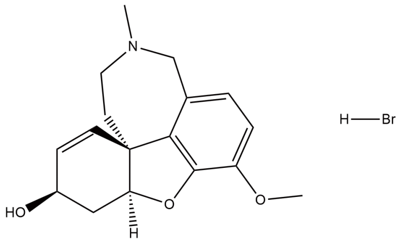

B5

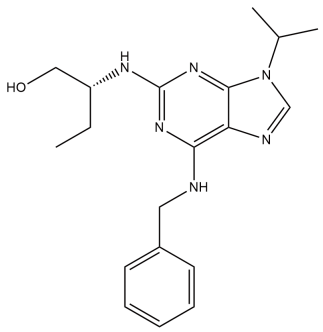

B6

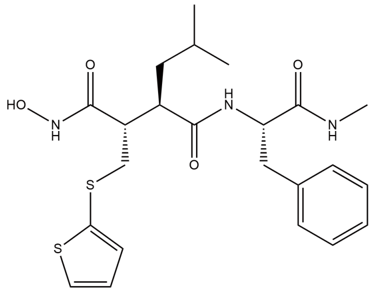

B7

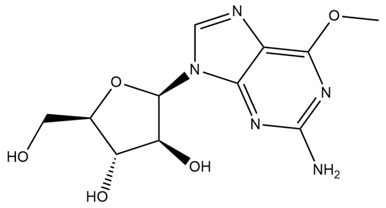

B8

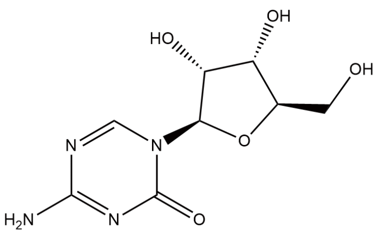

B9

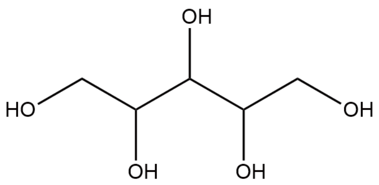

B10

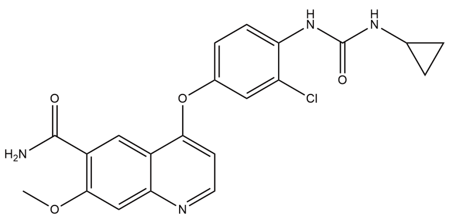

B11

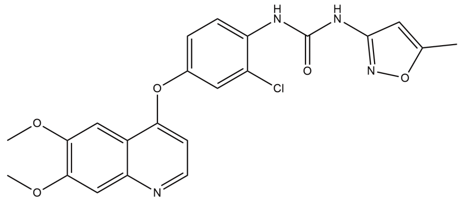

B12

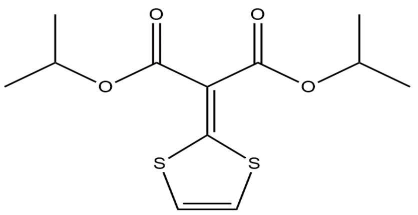

C1

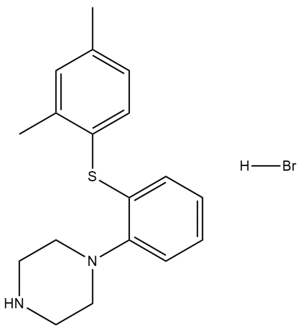

C2

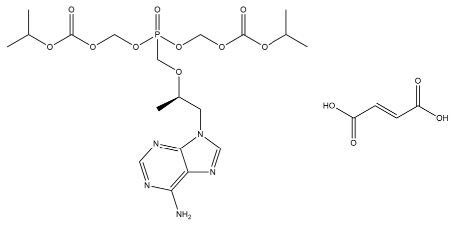

C3

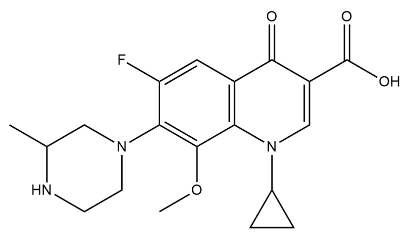

C4

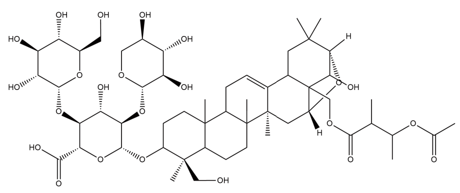

C5

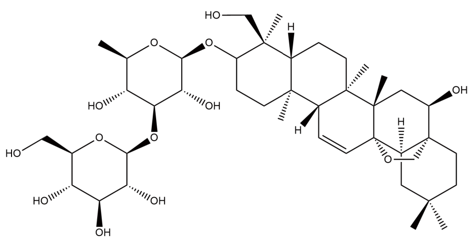

C6

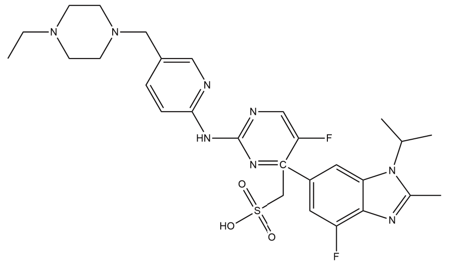

C7

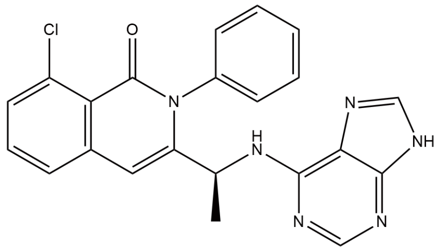

C8

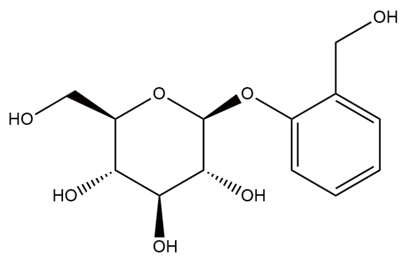

C9

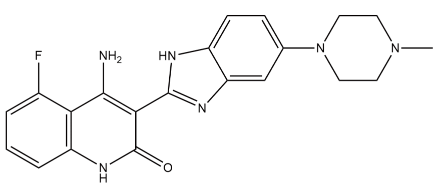

C10

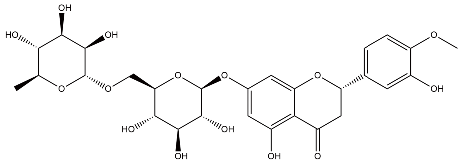

C11

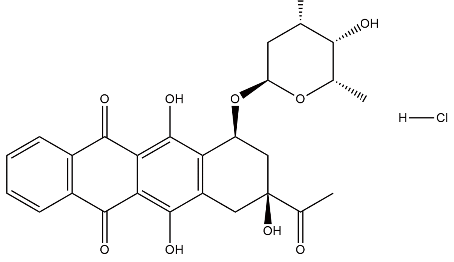

C12

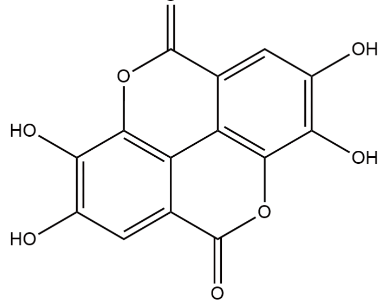

D1

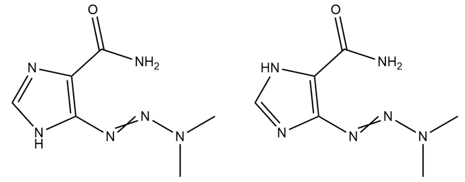

D2

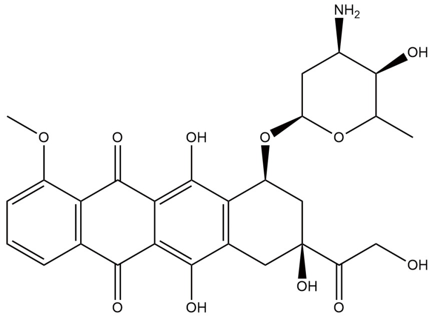

D3

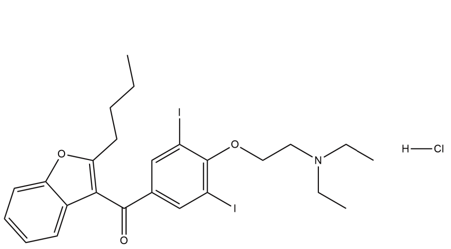

D4

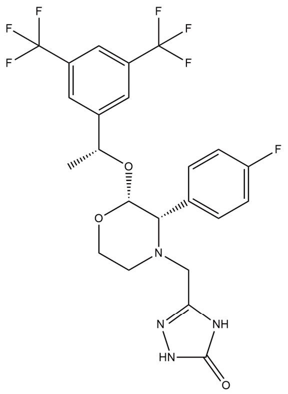

D5

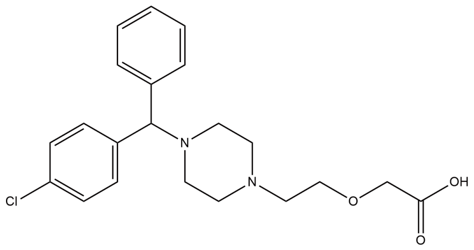

D6

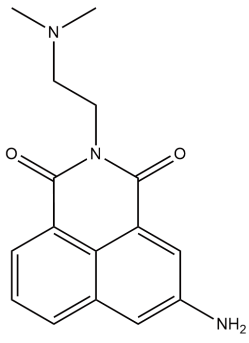

D7

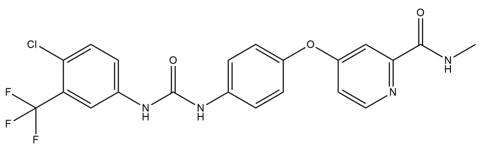

D8

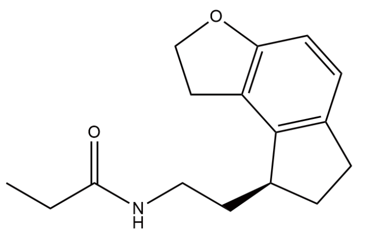

D9

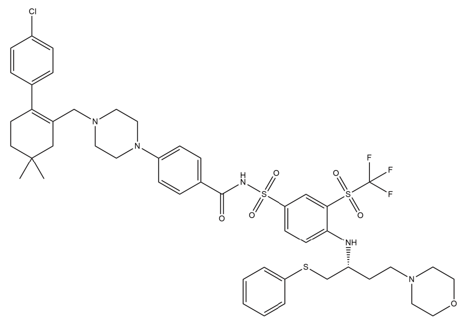

D10

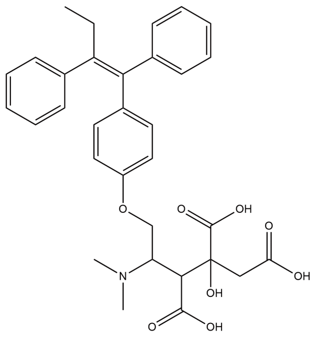

D11

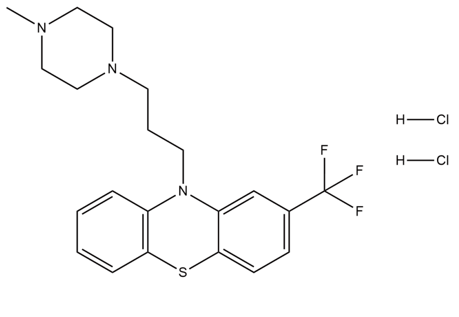

D12

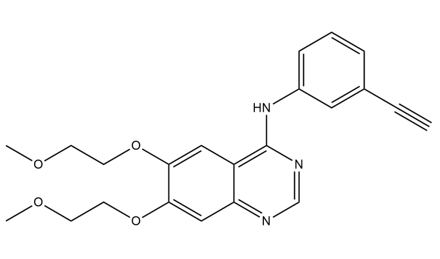

E1

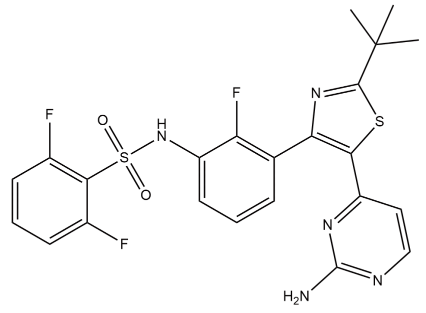

E2

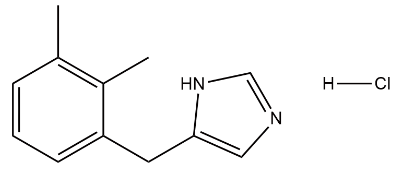

E3

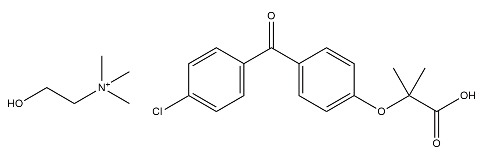

E4

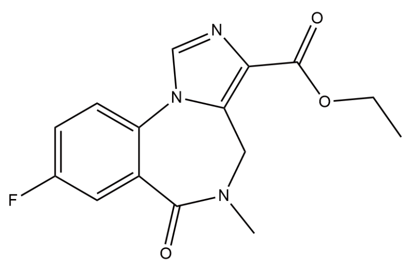

E5

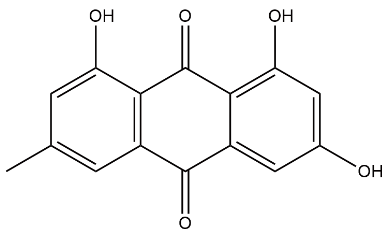

E6

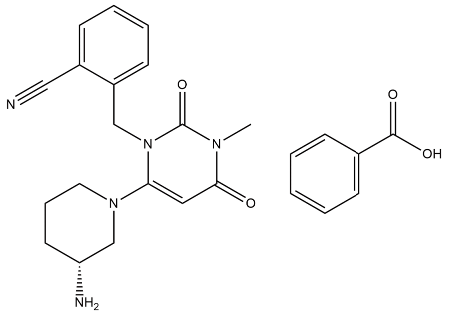

E7

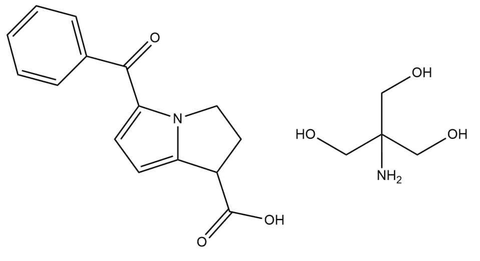

E8

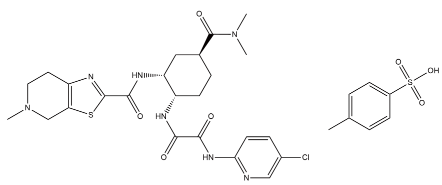

E9

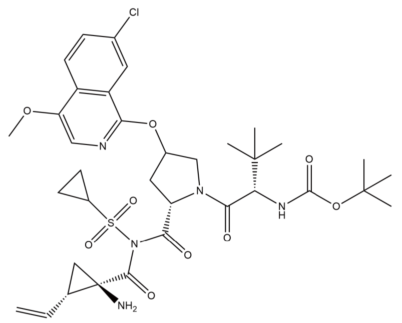

E10

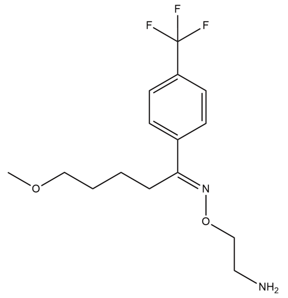

E11

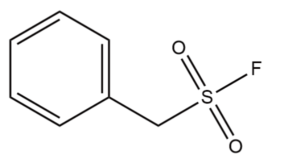

E12

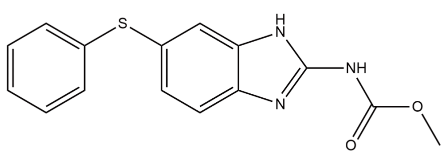

F1

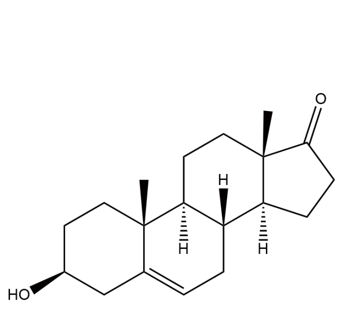

F2

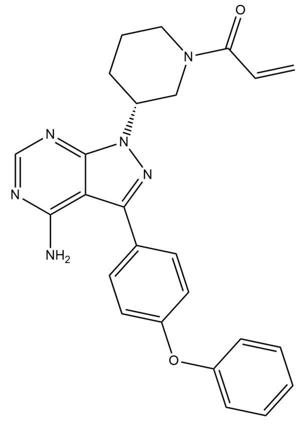

F3

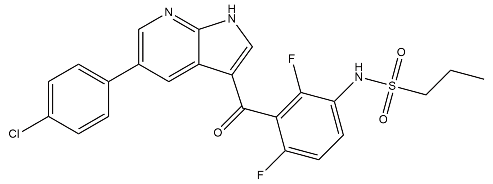

F4

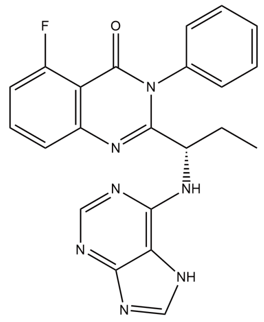

F5

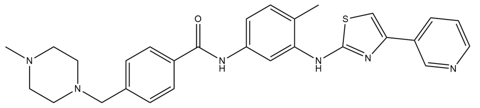

F6

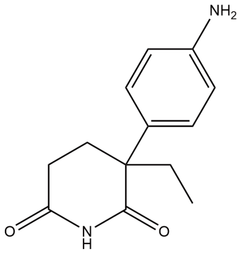

F7

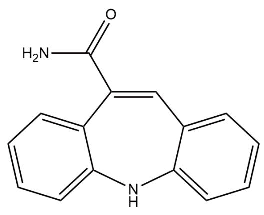

F8

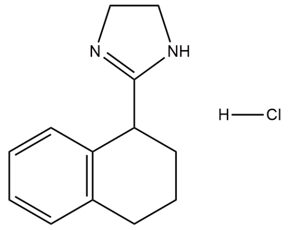

F9

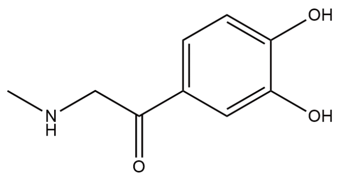

F10

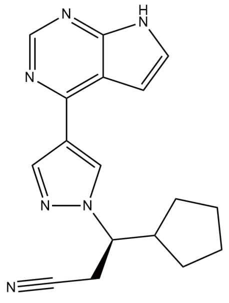

F11

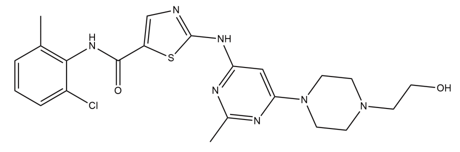

F12

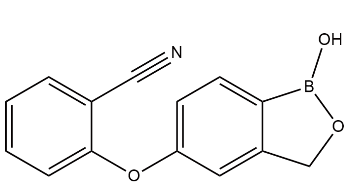

G1

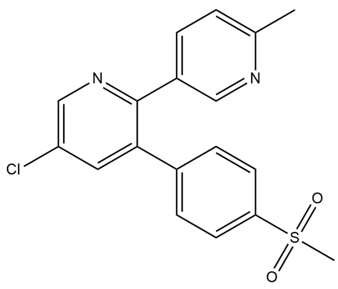

G2

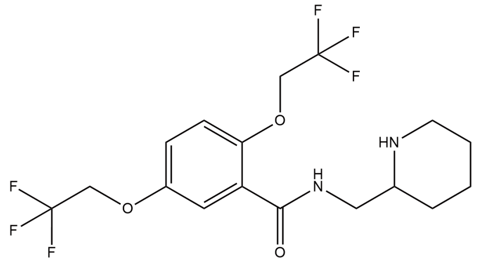

G3

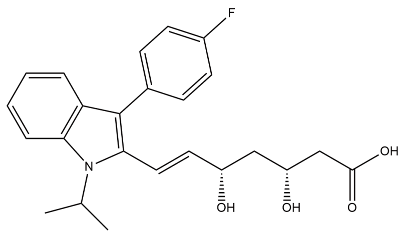

G4

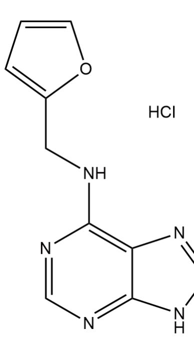

G5

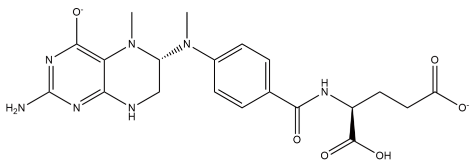

G6

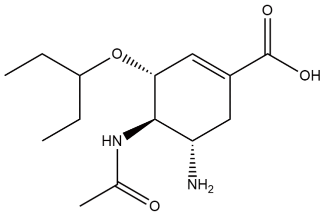

G7

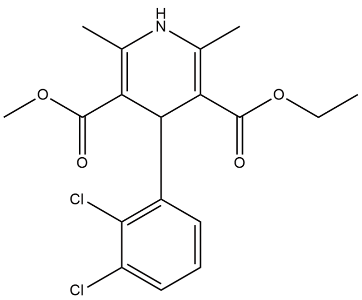

G8

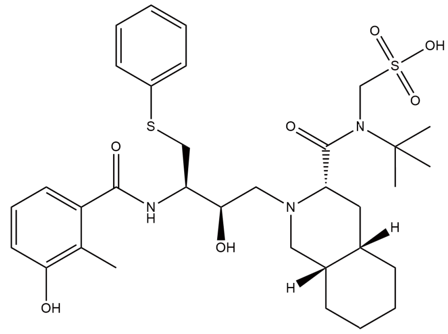

G9

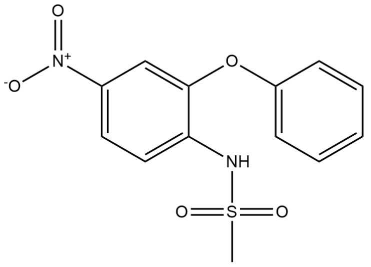

G10

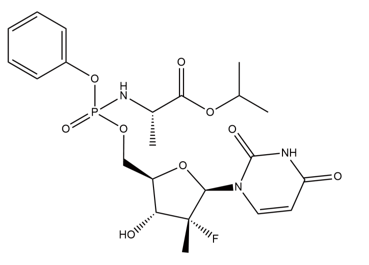

G11

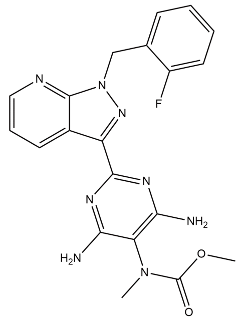

G12

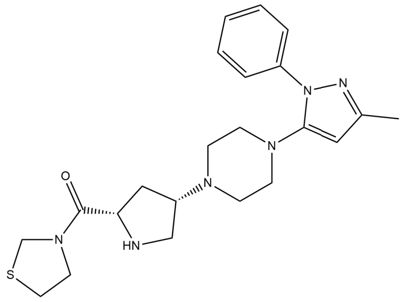

H1

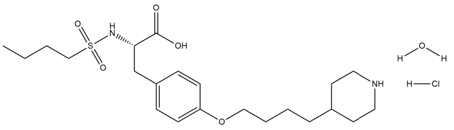

H2

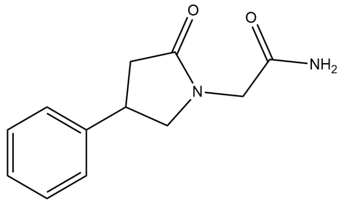

H3

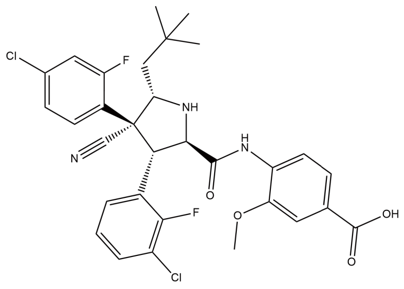

H4

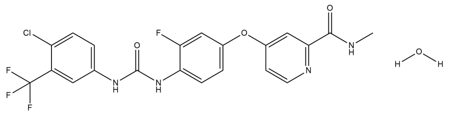

H5

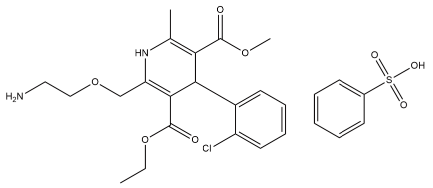

H6

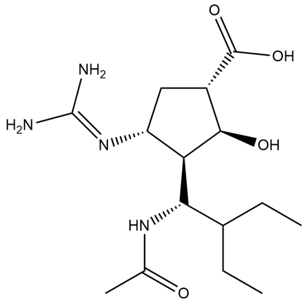

H7

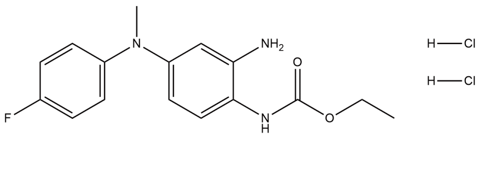

H8

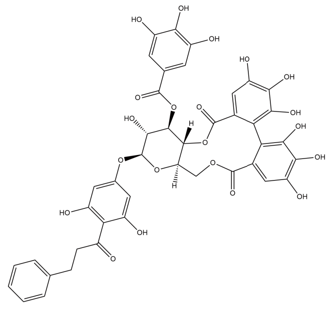

H9

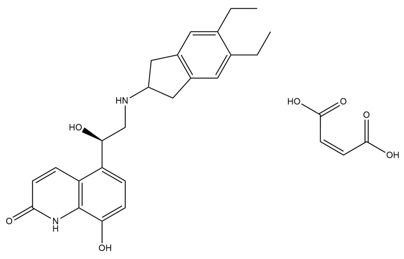

H10

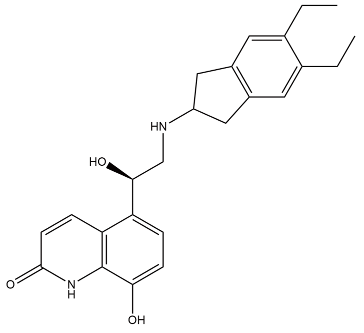

H11

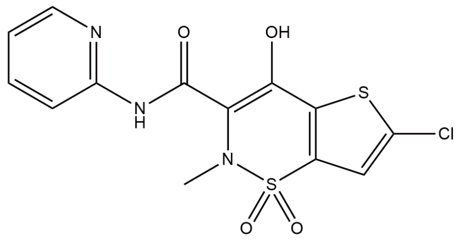

H12

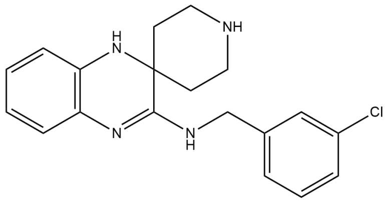

Figure S1. The chemical structures of the compounds used for screening ferroptosis inhibitors in RSL-3-induced PC-12 cells are shown below. The chemical names of these compounds are listed in Table S1.

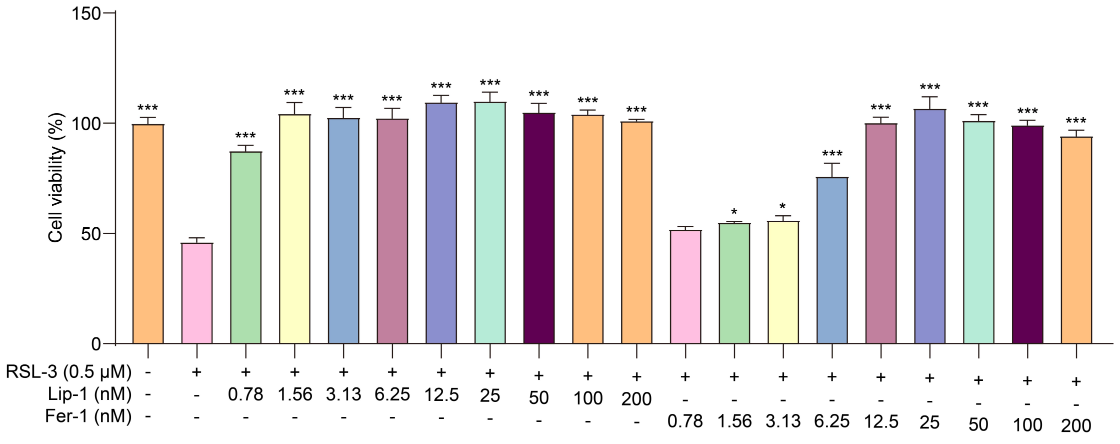

Figure S2. The effect of Lip-1 and Fer-1 at specified concentrations on the cell viability of RSL-3-induced PC-12 cells. The bar chart indicates the cell viability of PC-12 cells measured by MTT assay. Bar, SD. *, $p < 0.05$; **, $p < 0.01$; ***, $p < 0.001$.

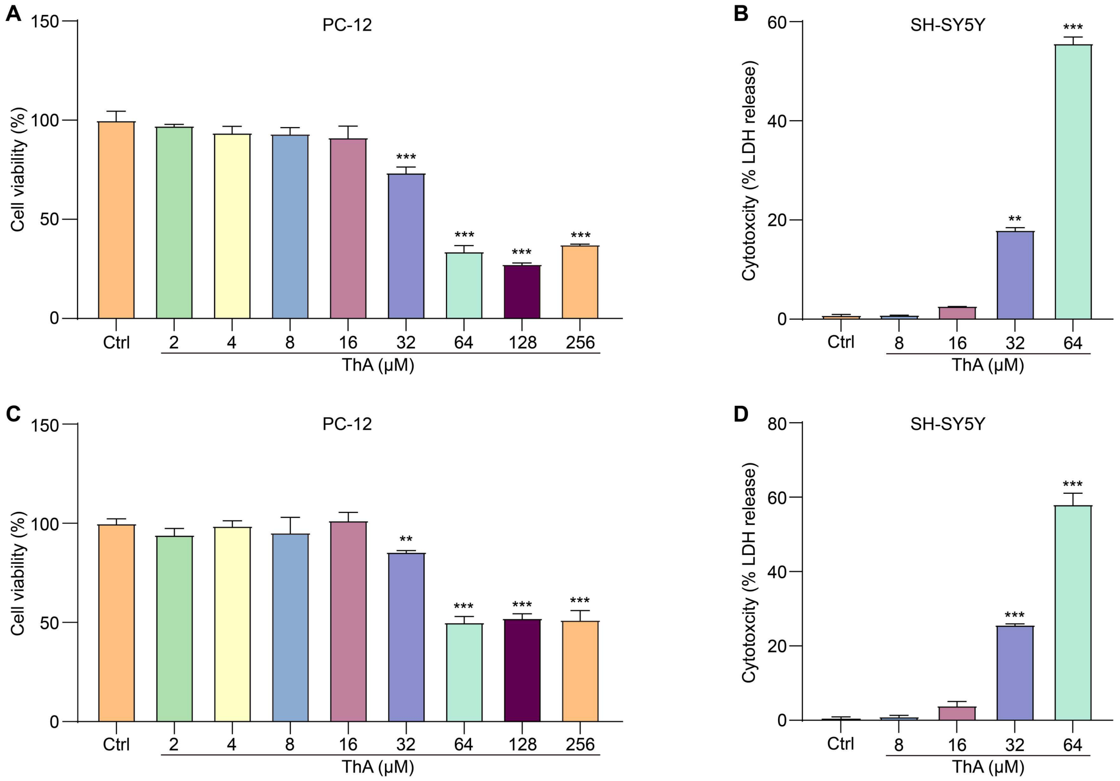

Figure S3. Evaluation of cell viability and cytotoxicity using MTT and LDH assays. (A) and (C) show the percentage of cell viability in PC-12 and SH-SY5Y cells treated with varying concentrations of ThA (2, 4, 8, 16, 32, 64, 128, 256 μM) as measured by the MTT assay. (B) and (D) display the cytotoxicity in PC12 and SH-SY5Y cells at the same ThA concentrations, as assessed by LDH release. Bar, SD. **, $p < 0.01$; ***, $p < 0.001$.

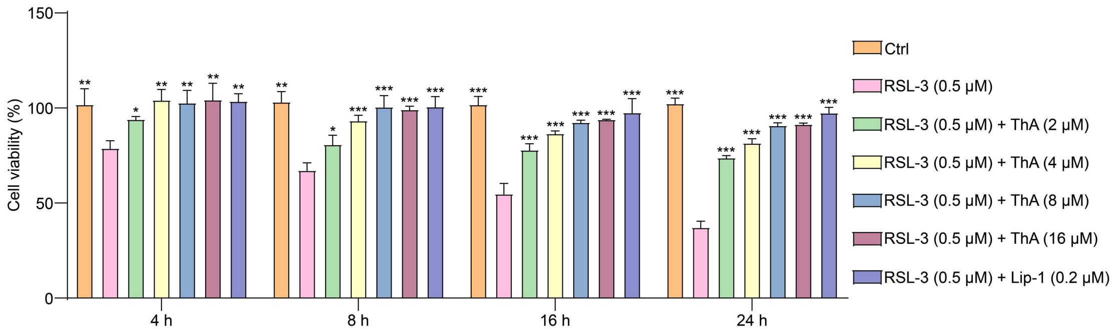

Figure S4. ThA increases the viability of PC-12 cells treated with RSL-3 at 4, 8, 16, and 24 h. The bar chart indicates the cell viability of PC-12 cells measured by MTT assay. Bar, SD. *, $p < 0.05$; **, $p < 0.01$; ***, $p < 0.001$.

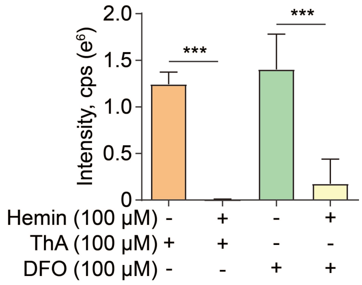

Figure S5. UHPLC-DAD-Q/TOF-MS/MS analysis of ThA and DFO in the samples, including ThA, DFO, ThA + hemin, and DFO + hemin group. The bar chart indicates the peak intensity of ThA and DFO in these samples. Bar, SD. *, $p < 0.05$; **, $p < 0.01$; ***, $p < 0.001$.

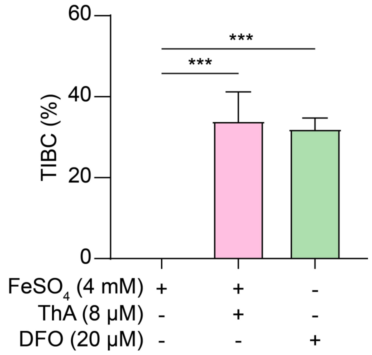

Figure S6. The iron chelating ability of ThA and DFO. The bar chart indicates the TIBC of ThA and DFO measured by the TIBC assay kit. Bar, SD. *, $p < 0.05$; **, $p < 0.01$; ***, $p < 0.001$.

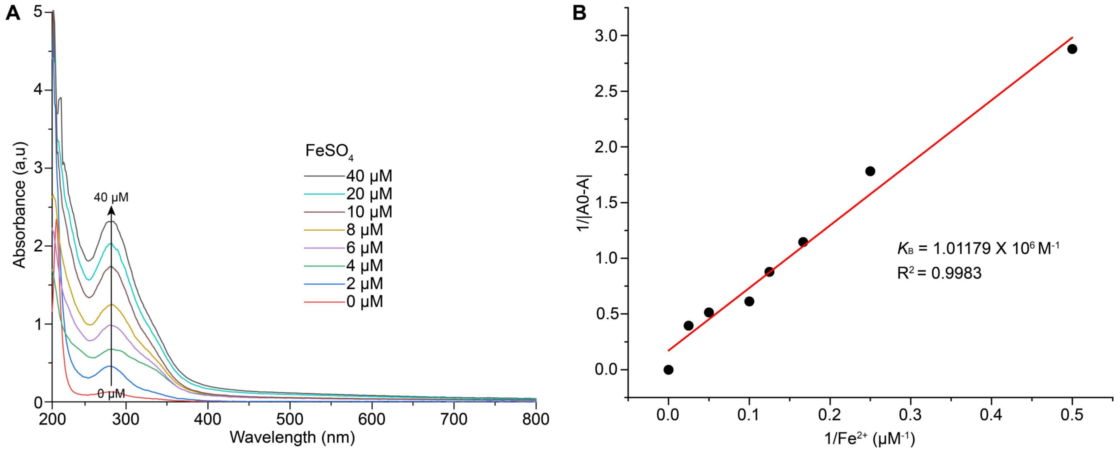

Figure S7. Absorption spectra of ThA in the presence of Fe^{2+} at various concentrations (0, 2, 4, 6, 8, 10, 20, 40 μM). (A) The absorbance was measured across a wavelength range of 200-800 nm, showing changes in the spectral profile as the concentration of Fe^{2+} increases. (B) Double reciprocal plot of $1/(A_0\text{-}A)$ versus $1/[Fe^{2+}]$ based on the absorption data. The linear fit provides a binding constant K_B=1.01179×10^6 M^{-1} and a correlation coefficient R^2=0.9983.

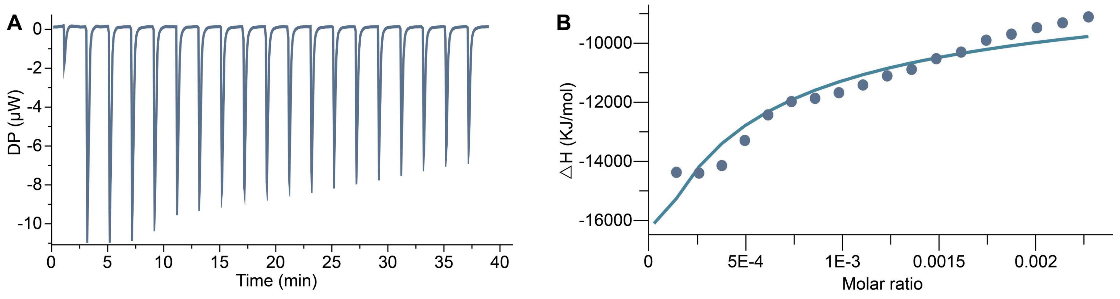

Figure S8. Isothermal titration calorimetry (ITC) analysis of the binding interaction between Fe^{2+} and ThA. (A) The raw ITC data showing the heat flow (DP) as a function of time during sequential injections of Fe^{2+} into the ThA solution. (B) The binding isotherm derived from the ITC data, where the change in enthalpy (ΔH) is plotted against the molar ratio of Fe^{2+} to ThA. The data were fitted to a binding model to calculate thermodynamic parameters of the interaction.

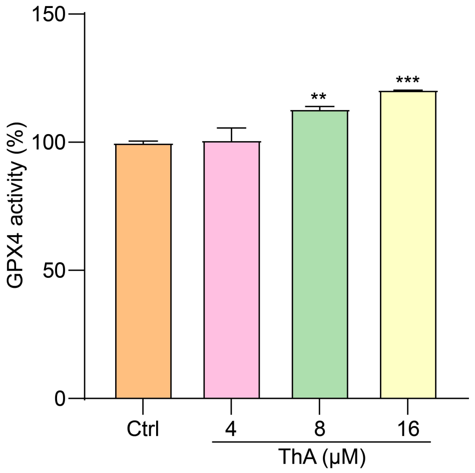

Figure S9. Measurement of GPX4 enzyme activity using the GPX4 Inhibitor Screening Assay Kit. The bar graph shows the relative GPX4 activity induced by ThA at concentrations of 4, 8, and 16 μM compared to the Ctrl. Bar, SD. **, $p < 0.01$; ***, $p < 0.001$.

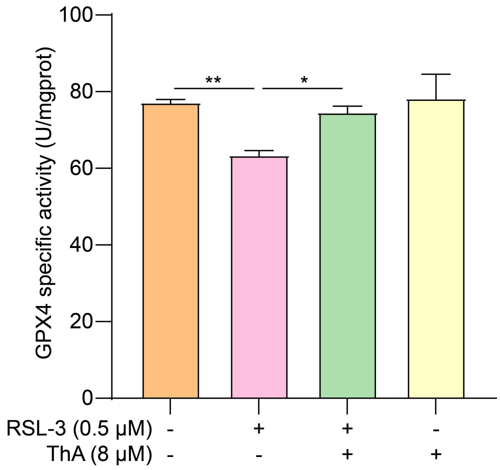

Figure S10. Measurement of GPX4 specific activity in PC12 cells using the Glutathione Peroxidase 4 Assay Kit. The bar graph shows GPX4 activity (U/mg protein) in cells under different treatment conditions: Ctrl, RSL-3 (0.5 μM) alone, RSL-3 combined with ThA (8 μM), and ThA (8 μM) alone. Bar, SD. *, $p < 0.05$; **, $p < 0.01$.

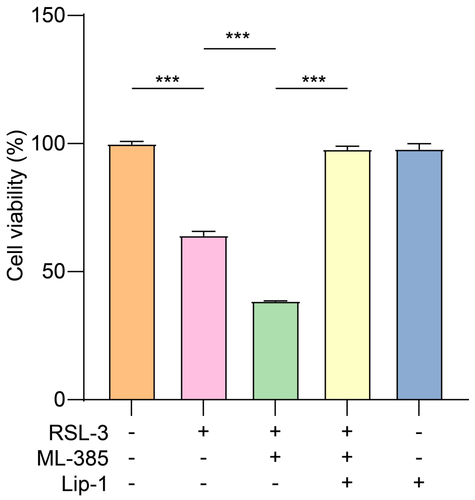

Figure S11. Effect of RSL-3, ML-385, and Lip-1 on cell viability. The bar graph shows the percentage of viability in PC-12 cells under different treatment conditions: Ctrl, RSL-3 (0.5 μM) alone, RSL-3 combined with ML-385 (4 μM), and RSL-3 combined with Lip-1 (0.2 μM). Bar, SD. ***, $p < 0.001$.

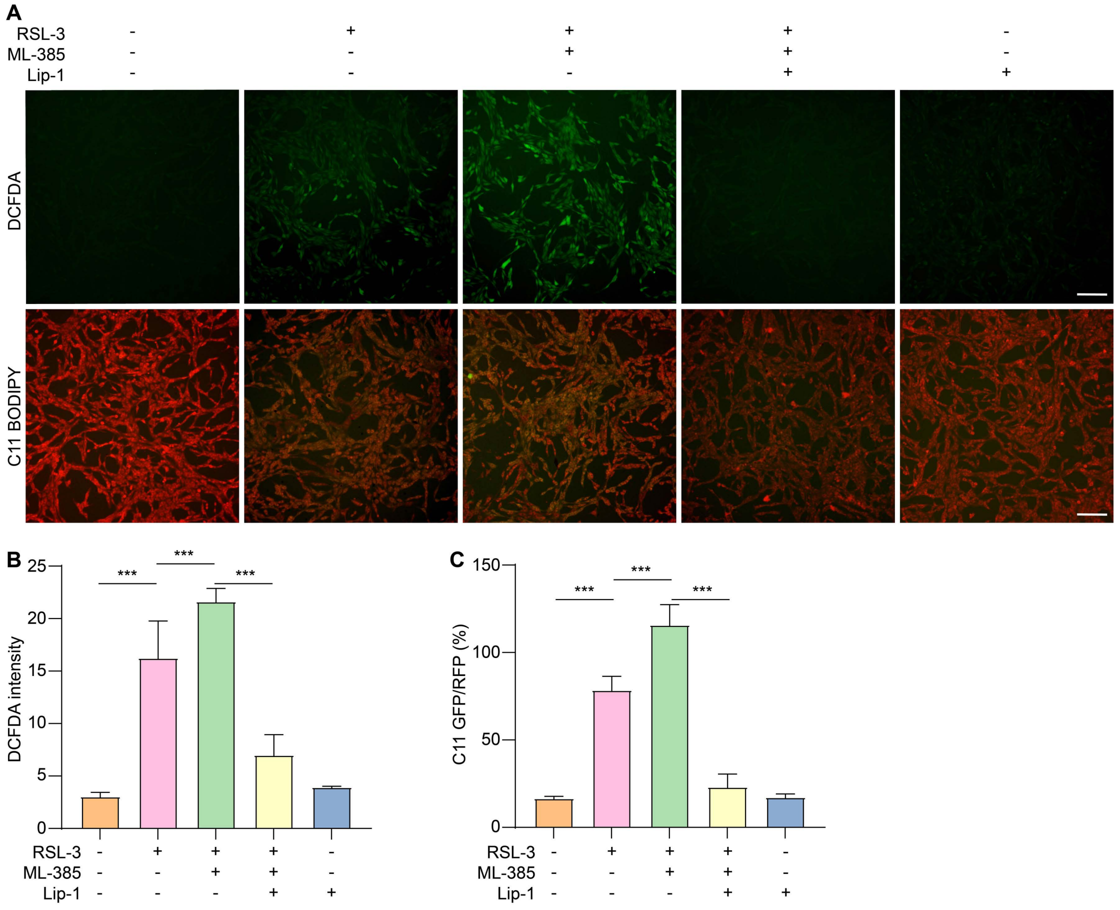

Figure S12. Evaluation of oxidative stress and lipid peroxidation in PC-12 cells treated with RSL-3, ML-385, and Lip-1. (A) Representative fluorescence images showing

ROS levels detected by DCFDA (top row) and lipid peroxidation detected by C11 BODIPY (bottom row) under different treatment conditions. Scale bars: 200 μm. (B) Quantification of DCFDA fluorescence intensity, reflecting intracellular ROS levels. (C) Quantification of C11 BODIPY fluorescence intensity ratio (GFP/RFP), indicating the level of lipid peroxidation. Bar, SD. ***, $p < 0.001$.

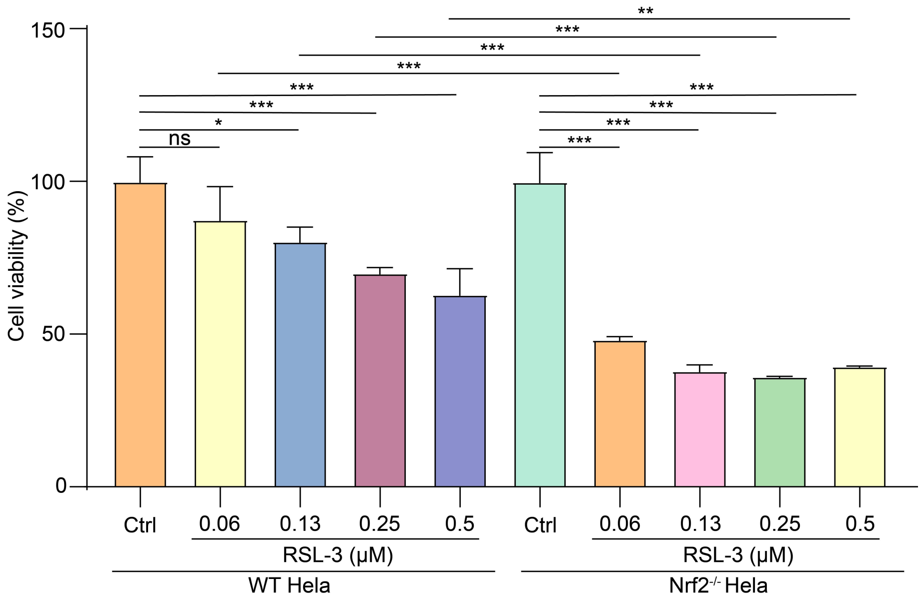

Figure S13. Cell viability assessment in WT and Nrf2$^{-/-}$ HeLa cells treated with different concentrations of RSL-3, measured by the MTT assay. The bar graph shows the percentage of cell viability after treatment with increasing concentrations of RSL-3 (0.06, 0.13, 0.25, 0.5 μM) compared to the Ctrl in both WT and Nrf2$^{-/-}$ HeLa cells. Bar, SD. *, $p < 0.05$; **, $p < 0.01$; ***, $p < 0.001$.

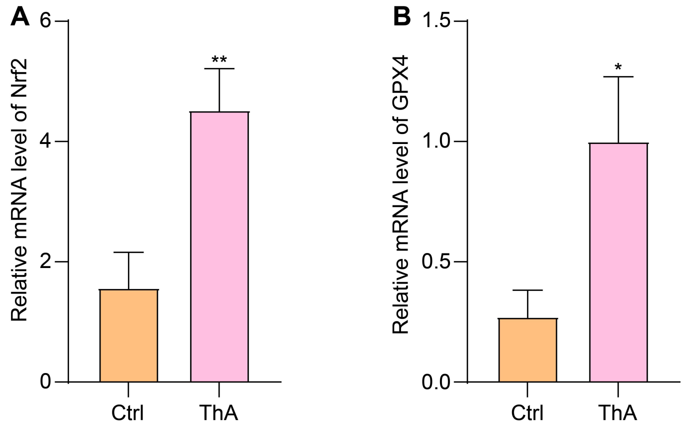

Figure S14. Relative mRNA expression levels of Nrf2 and GPX4 in PC-12 cells treated with ThA. (A) The bar graph shows the relative mRNA level of Nrf2 in Ctrl and ThA-treated cells. (B) The bar graph shows the relative mRNA level of GPX4 in Ctrl and ThA-treated cells. Bar, SD. *, $p < 0.05$; **, $p < 0.01$.

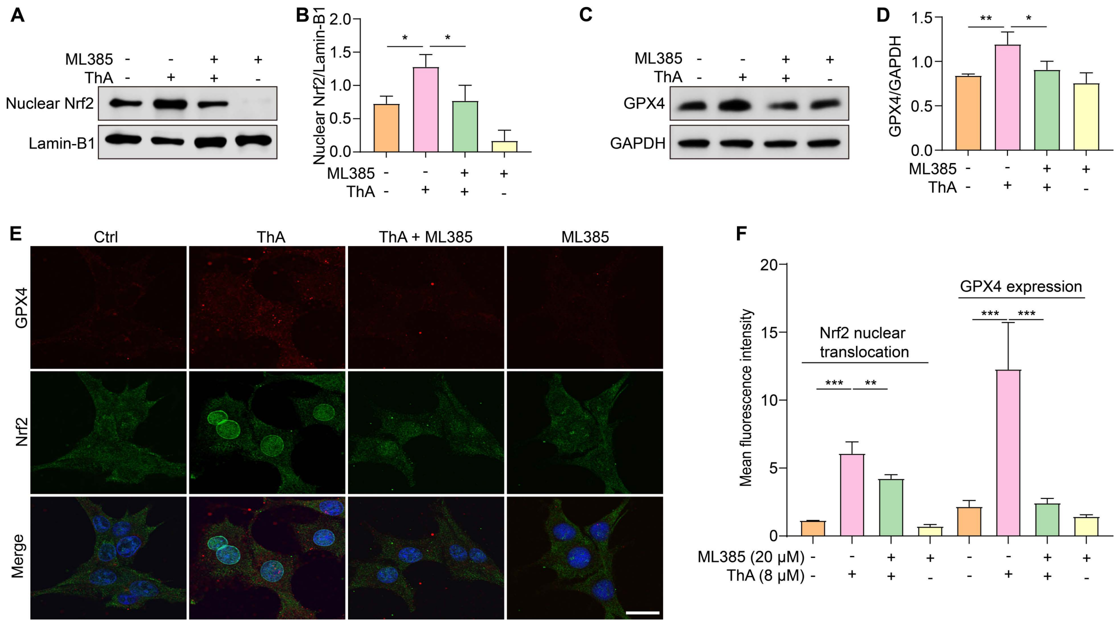

Figure S15. ML385 inhibits the nuclear translocation of Nrf2 and GPX4 expression in PC-12 cells. (A, C) Representative Western blot images of nuclear Nrf2, GPX3, Lamin-B1, and GAPDH in PC12 cells treated with 8 μM ThA in the presence or absence of 20 μM ML385. Full-length Western blot images are presented in Figure S36. (B, D) Bar charts indicate the ratios of nuclear Nrf2 to lamin-B1 and GPX4 to GAPDH. (E) Representative immunofluorescence images demonstrate Nrf2 nuclear translocation and GPX4 expression in PC-12 cells treated with 8 μM ThA in the presence or absence of 20 μM ML385. Magnification: 63×, Scale bars: 50 μm. (F) The bar chart indicates the mean fluorescence intensity of Nrf2 nuclear translocation and GPX4 expression in PC-12 cells. Bar, SD. *, $p < 0.05$; **, $p < 0.01$; ***, $p < 0.001$.

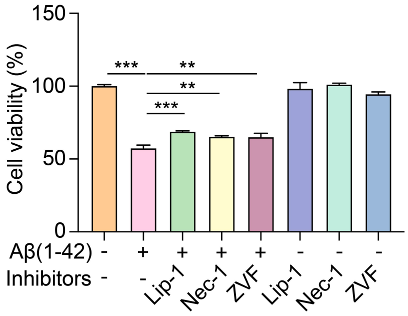

Figure S16. Aβ induces apoptosis, necrosis, and ferroptosis in PC-12 cells. The bar chart indicates the cell viability of PC-12 cells treated with 25 μM Aβ(1-42) in the presence or absence of 0.2 μM Lip-1, 10 μM Nec-1, and 2 μM ZVF. Bar, SD. *, $p < 0.05$; **, $p < 0.01$; ***, $p < 0.001$.

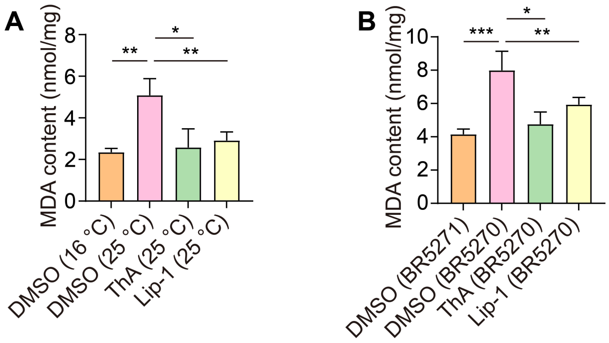

Figure S17. ThA reduces the MDA content in CL4176 and BR5270 worms. (A) The bar chart indicates the relative MDA content in CL4176 worms treated with or without 20 μM ThA and 200 μM Lip-1. (B) The bar chart indicates the relative MDA content in BR5271 and BR5270 worms treated with or without 20 μM ThA and 200 μM Lip-1. Bar, SD. *, $p < 0.05$; **, $p < 0.01$; ***, $p < 0.001$.

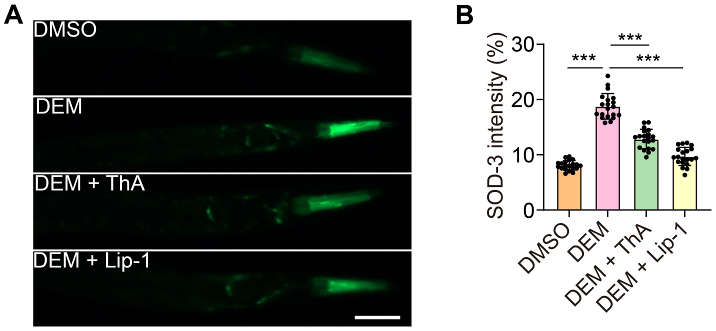

Figure S18. ThA reduces the SOD-3 intensity in CF1553 worms. (A) Representative images of CF1553 worms treated with 1 mM DEM in the presence or absence of 20

μM ThA and 200 μM Lip-1. Magnification: × 20; scale bar: 200 μm. (B) The bar charts indicate the relative SOD-3 intensity in CF1553 worms. Bar, SD. *, $p < 0.05$; **, $p < 0.01$; ***, $p < 0.001$.

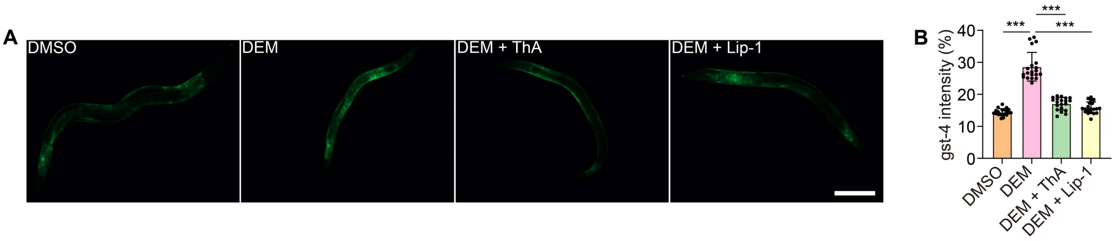

Figure S19. ThA reduces the gst-4 intensity in CL2166 worms. (A) Representative images of CL2166 worms treated with 1 mM DEM in the presence or absence of 20 μM ThA and 200 μM Lip-1. Magnification: × 20; scale bar: 200 μm. (B) The bar charts indicate the relative gst-4 intensity in CL2166 worms. Bar, SD. *, $p < 0.05$; **, $p < 0.01$; ***, $p < 0.001$.

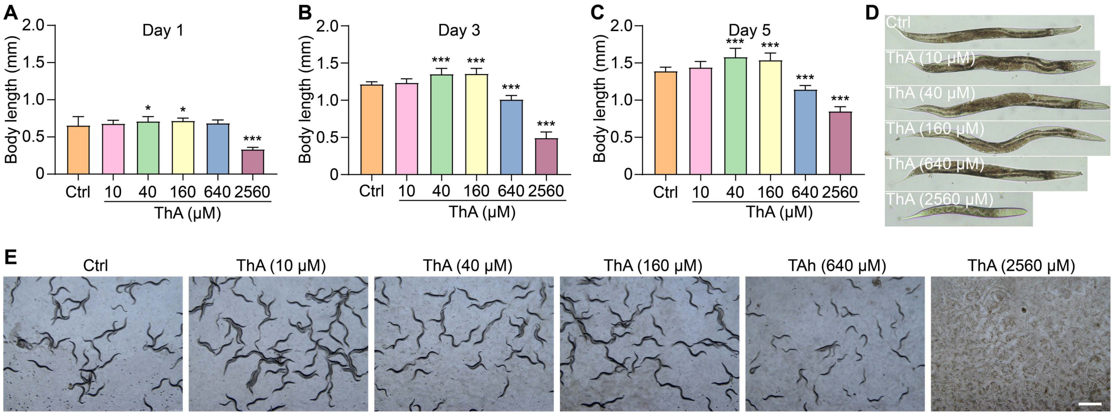

Figure S20. Effects of ThA on the body length and morphology of N2 *C. elegans*. (A-C) Bar graphs showing the body length of *C. elegans* on Day 1, Day 3, and Day 5 after exposure to different concentrations of ThA (10, 40, 160, 640, 2560 μM). (D) Representative images of individual *C. elegans* from each treatment group on Day 5, illustrating the effect of ThA on worm body length. Scale bar:100 μm. (E) Representative images of the population and morphology of *C. elegans* under each treatment condition. Scale bar:100 μm. Bar, SD. *, $p < 0.05$; **, $p < 0.01$; ***, $p < 0.001$.

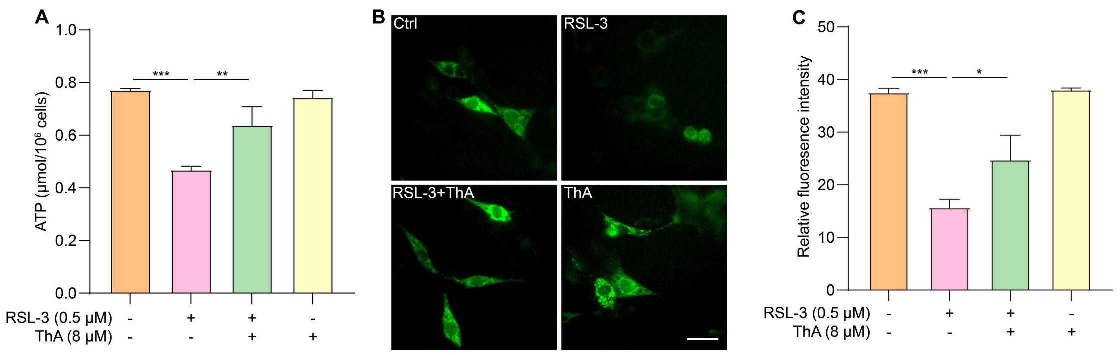

Figure S21. Assessment of intracellular ATP levels and mitochondrial ATP in PC-12 cells treated with RSL-3 in the presence or absence of ThA. (A) Bar graph showing ATP levels (μmol/10^6 cells) in different treatment groups: Ctrl, RSL-3 (0.5 μM), RSL-3 combined with ThA (8 μM), and ThA (8 μM) alone. (B) Representative fluorescence images showing mitochondrial ATP levels in PC-12 cells transfected with the pCMV-Mito-AT1.03 plasmid, observed using fluorescence microscopy. Scale bar: 100 μm. (C) Quantification of relative fluorescence intensity from the mitochondrial ATP sensor, indicating changes in mitochondrial ATP production. Bar, SD. *, $p < 0.05$; **, $p < 0.01$; ***, $p < 0.001$.

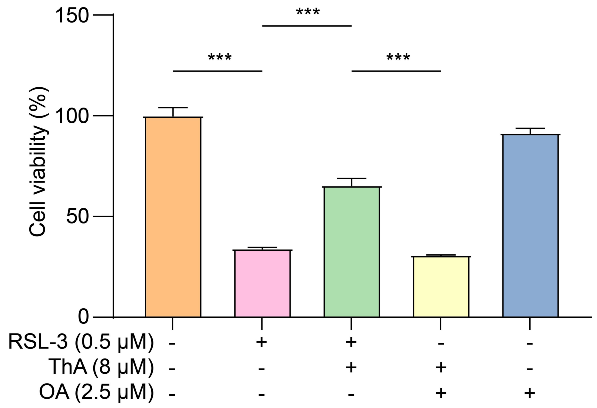

Figure S22. Effect of RSL-3, ThA, and OA on cell viability. The bar graph shows the percentage of cell viability under different treatment conditions: Ctrl, RSL-3 (0.5 μM) alone, RSL-3 combined with ThA (8 μM), RSL-3 combined with OA (2.5 μM), and OA alone. Bar, SD. ***, $p < 0.001$.

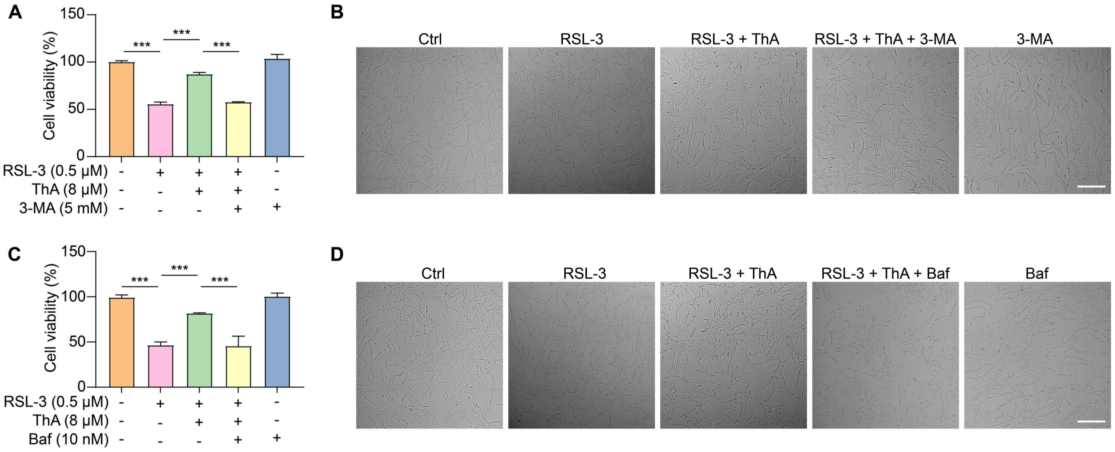

Figure S23. Autophagy inhibitors 3-MA and Baf reverse the inhibitory effect of ThA on the cell death of RSL-3-induced PC-12 cells. (A, C) Bar charts indicate the cell viability of RSL-3-induced PC-12 cells, treated with ThA in the presence or absence of 3-MA and Baf at specified concentrations. Bar, SD. *, $p < 0.05$; **, $p < 0.01$; ***, $p < 0.001$. (B, D) Representative images of RSL-3-induced PC-12 cells, treated with ThA in the presence or absence of 3-MA and Baf at specified concentrations. Magnification: 10×, Scale bars: 200 μm.

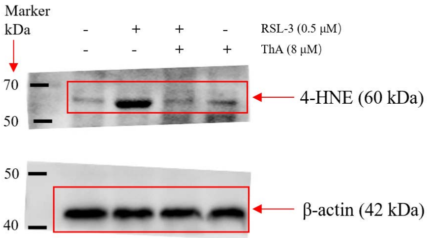

Figure S24. Full-length Western blotting images of Figure 1P

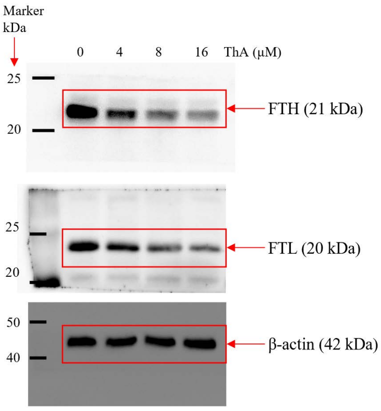

Figure S25. Full-length Western blotting images of Figure 2P

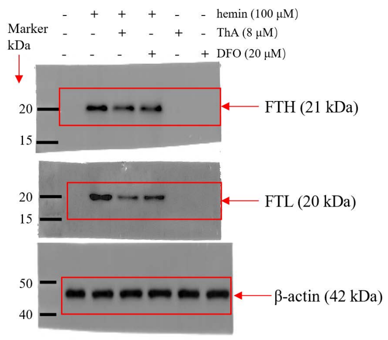

Figure S26. Full-length Western blotting images of Figure 2S

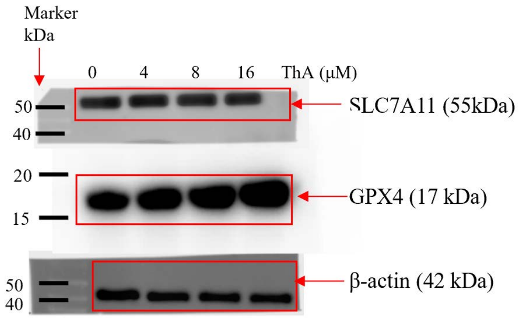

Figure S27. Full-length Western blotting images of Figure 3A

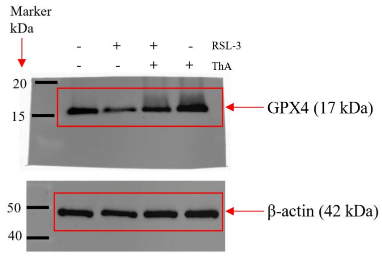

Figure S28. Full-length Western blotting images of Figure 3D

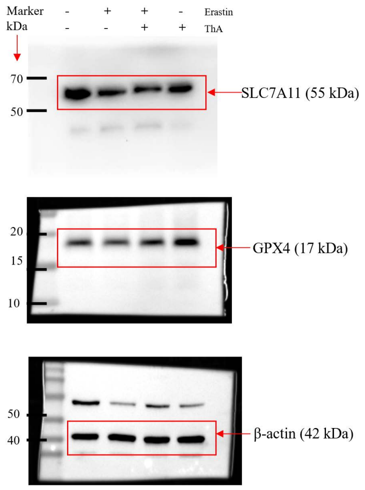

Figure S29. Full-length Western blotting images of Figure 3F

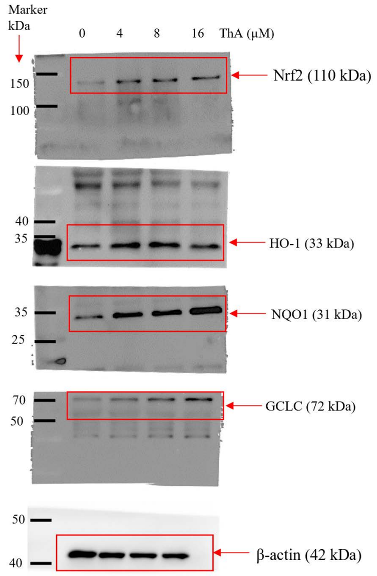

Figure S30. Full-length Western blotting images of Figure 4A

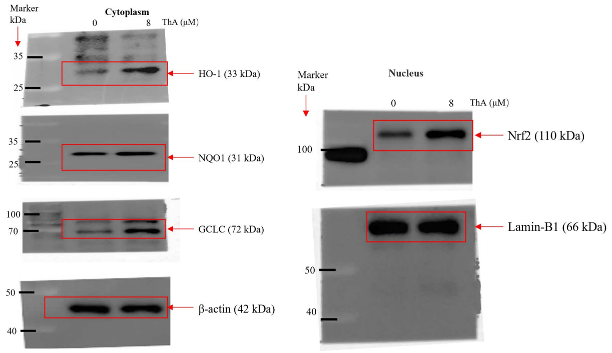

Figure S31. Full-length Western blotting images of Figure 4F

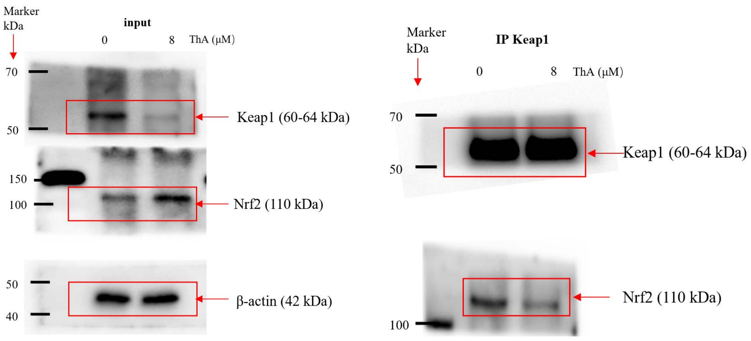

Figure S32. Full-length Western blotting images of Figure 4K

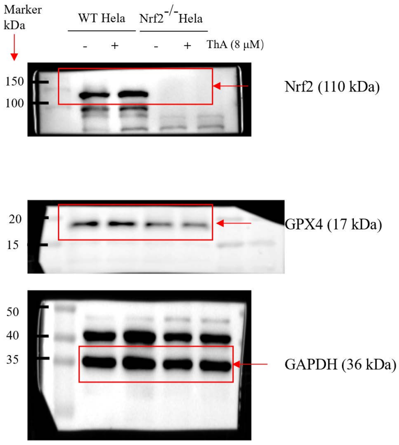

Figure S33. Full-length Western blotting images of Figure 5L

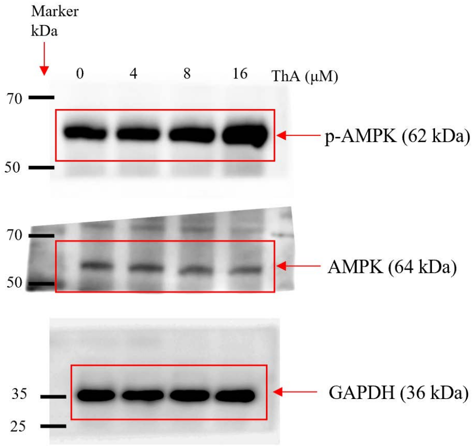

Figure S34. Full-length Western blotting images of Figure 6A

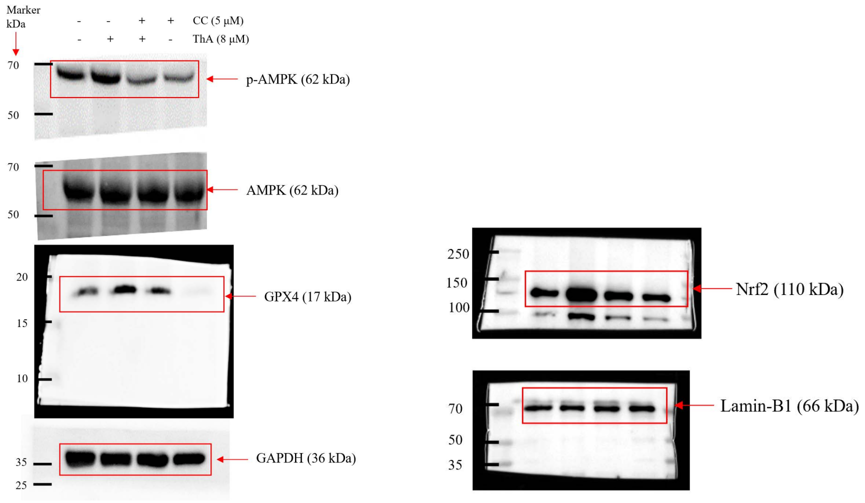

Figure S35. Full-length Western blotting images of Figure 6C

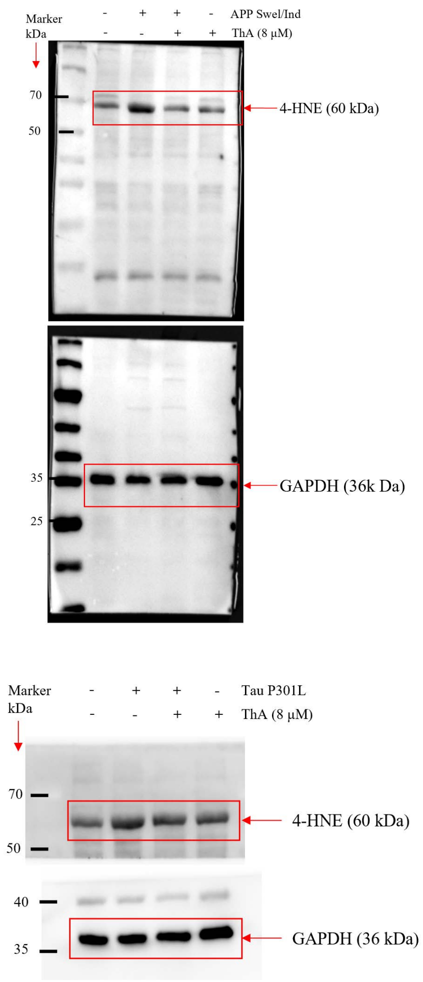

Figure S36. Full-length Western blotting images of Figure 7I and K

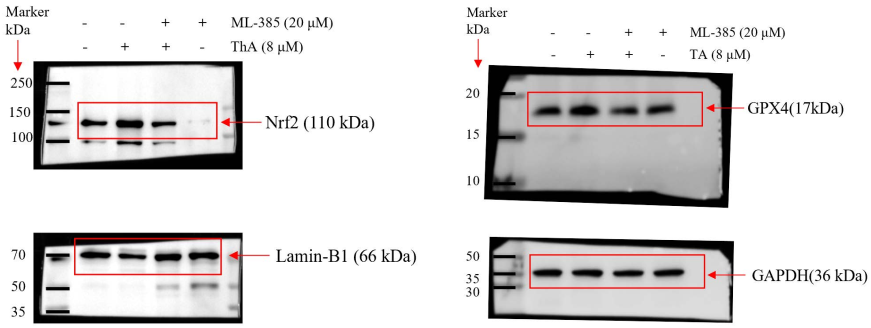

Figure S37. Full-length Western blotting images of Figure S15A and C

Table S1. The compounds used for screening ferroptosis inhibitors in RSL-3-induced PC-12 cells.

A1<br>MEK162	A2<br>Lidocaine	A3<br>Prasugrel	A4<br>Angiotensin II	A5<br>Daptomycin	A6<br>Rigosertib	A7<br>Levetiracetam	A8<br>MEK162	A9<br>Decitabine	A10<br>Macitentan	A11<br>Doripenem hydrate	A12<br>Gemcitabine HCl
B1<br>Imatinib mesylate	B2<br>Carboplatin	B3<br>Vatalanib (PTK787) 2HCl	B4<br>Galanthamine HBr	B5<br>Roscovitine	B6<br>Batimastat	B7<br>Nelarabine	B8<br>5-Azacytidine	B9<br>Xylitol	B10<br>Lenvatinib	B11<br>Tivozanib	B12<br>Malotilate
C1<br>Vortioxetine HBr	C2<br>Tenofovir disoproxil fumarate	C3<br>Gatifloxacin	C4<br>Sodium aescinate	C5<br>Saikosaponin A	C6<br>LY2835219	C7<br>IPI-145	C8<br>D-(-)-Salicin	C9<br>Dovitinib	C10<br>Idarubicin HCl	C11<br>Idarubicin HCl	C12<br>Ellagic acid
D1<br>Dacarbazine	D2<br>Epirubicin HCl	D3<br>Amiodarone HCl	D4<br>Aprepitant	D5<br>Cetirizine	D6<br>Amonafide	D7<br>Sorafenib	D8<br>ABT-263	D9<br>Cinacalcet HCl	D10<br>Tamoxifen citrate	D11<br>Trifluoperazine 2HCl	D12<br>Erlotinib
E1<br>Dabrafenib mesylate	E2<br>Detomidine HCl	E3<br>Choline fenofibrate	E4<br>Flumazenil	E5<br>Emodin	E6<br>Alogliptin benzoate	E7<br>Ketorolac tromethamine salt	E8<br>Edoxaban tosylate monohydrate	E9<br>Asunaprevir	E10<br>Fluvoxamine maleate	E11<br>Phenylmethylsul fonyl fluoride	E12<br>Fenbendazole
F1<br>Dehydroepiandr osterone	F2<br>Ibrutinib	F3<br>Vemurafenib	F4<br>Idelalisib	F5<br>Masitinib	F6<br>Aminoglutethimi de	F7<br>Carbamazepine	F8<br>Tetrahydrozoline HCl	F9<br>Adrenalone HCl	F10<br>Ruxolitinib	F11<br>AN-2728	F12<br>Tedizolid
G1<br>Etoricoxib	G2<br>Flecainide acetate	G3<br>Fluvastatin	G4<br>Kinetin	G5<br>Levomefolate calcium	G6<br>Oseltamivir	G7<br>Oseltamivir acid	G8<br>Potassium canrenoate	G9<br>Nimesulide	G10<br>Sofosbuvir	G11<br>Riociguat	G12<br>Teneligliptin hydrobromide
H1<br>Tirofiban hydrochloride monohydrate	H2<br>Phenylpiracetam	H3<br>RG7388	H4<br>Regorafenib monohydrate	H5<br>Amlodipine Besylate	H6<br>Peramivir	H7<br>Retigabine dihydrochloride	H8<br>Thonningianin A	H9<br>Salmeterol xinafoate	H10<br>Indacaterol maleate	H11<br>Lornoxicam	H12<br>Liproxstatin-1
